# Realizing acoustic qubit analogues with nonlinearly tunable phi-bits in externally driven coupled acoustic waveguides

**DOI:** 10.1038/s41598-023-27427-4

**Published:** 2023-01-12

**Authors:** P. A. Deymier, K. Runge, M. A. Hasan, T. D. Lata, J. A. Levine, P. Cutillas

**Affiliations:** 1 Department of Materials Science and Engineering, University of Arizona, Tucson, AZ 85721 USA; 2 Department of Mechanical Engineering, Wayne State University, Detroit, MI 48202 USA; 3 Department of Computer Science, The University of Arizona, Tucson, AZ 85721 USA

**Keywords:** Computer science, Information theory and computation

## Abstract

Using experiments and theory, we investigate the behavior of nonlinear acoustic modes in a physical system composed of an array of three coupled acoustic waveguides, two of which are externally driven with two different frequencies. Nonlinear modes with frequency given by linear combinations of the driving frequencies are realizations of so-called logical phi-bits. A phi-bit is a two-state degree of freedom of an acoustic wave, which can be in a coherent superposition of states with complex amplitude coefficients, i.e., a qubit analogue. We demonstrate experimentally that phi-bit modes are supported in the array of waveguides. Using perturbation theory, we show that phi-bits may result from the intrinsic nonlinearity of the material used to couple the waveguides. We have also isolated possible effects on phi-bit states associated with the systems’ electronics, transducers and ultrasonic coupling agents used to probe the array and that may introduce extrinsic nonlinearities. These extrinsic effects are shown to be easily separable from the intrinsic behavior. The intrinsic behavior includes sharp jumps in phi-bit phases occurring over very narrow ranges of driving frequency. These jumps may also exhibit hysteretic behavior dependent on the direction of driving frequency tuning. The intrinsic states of phi-bits and multiple nonlinearly correlated phi-bits may serve as foundation for robust and practical quantum-analogue information technologies.

## Introduction

Quantum computing is essentially phase computing; it exploits the possibility of achieving and rotating the coherent superpositions of states of correlated multipartite systems with complex amplitudes that are represented as vectors in large, exponentially complex Hilbert spaces. The notion of “classical entanglement” for sound waves possesses the non-separability and complexity essential to reach the promise of parallelism in quantum computing, yet without the fragility of decoherence even at room temperature. In a previous publication, we have shown that one can achieve non-separable acoustic waves as classical analogues of quantum non-separable states^1^ and we introduced the concept of “phi-bits”^2^. A phi-bit is a two-state degree of freedom of an acoustic wave (the acoustic spin), which can be in a coherent superposition of states with complex amplitude coefficients. Hence, a phi-bit is a qubit classical analogue—the critical component of quantum information science (QIS) platforms. Theoretically, computationally, and experimentally, we demonstrated the exponentially complex and scalable Hilbert space of states of multiple logical phi-bits (≤ 16 with 2^16^ dimensional space) and the non-separability of coherent superpositions to reveal their applicability to QIS^3^. The use of phi-bits as qubit analogues for the development of quantum-analogue computing platforms that can support and navigate scalable, exponentially complex Hilbert spaces is aided by an understanding of the nonlinear phenomena that enable phi-bits as well as their correlations. Indeed, navigation of multiple correlated phi-bit Hilbert spaces to achieve operations analogous to quantum gates necessitate predictability in the phi-bit response to parametric changes in the physical system that support them. The objective of the present paper is to shed light on the origin of phi-bit behavior in a physical system composed of an externally driven array of acoustic waveguides. In particular, we focus on identifying the intrinsic nonlinearities in this physical system that lead to predictable changes in the coherent state of phi-bits upon a change in the driving conditions. We examine an array of waveguide that is driven with two different frequencies whereby phi-bits are realized as nonlinear modes with frequencies that are linear combinations of the driving frequencies. In Section “Physical system, logical phi-bits and sources of nonlinearity”, we describe in detail the phi-bit-supporting physical system as well as the possible intrinsic and extrinsic sources of nonlinearity. Experimental results are presented in Section “Experimental results”. These results focus on the behavior of the phase of the phi-bit state as one of the two driving frequency is tuned. Sections “Possible origin of the phase backgrounds” and “Possible origin of the π jumps” propose theoretical models of the phi-bit-supporting system that predict quantitatively and/or qualitatively experimentally observed behaviors. These models offer a window on the possible origin of sources of nonlinearity that underlie the observed behaviors. In Section “Predictable quantum-like operations using phase jumps” we illustrate how the nonlinear response of two phi-bits may be used to operate on them. Finally in Section “Conclusions”, we draw conclusions on the predictability of phi-bit behavior and their applicability to QIS.

## Physical system, logical phi-bits and sources of nonlinearity

**Figure 1:**
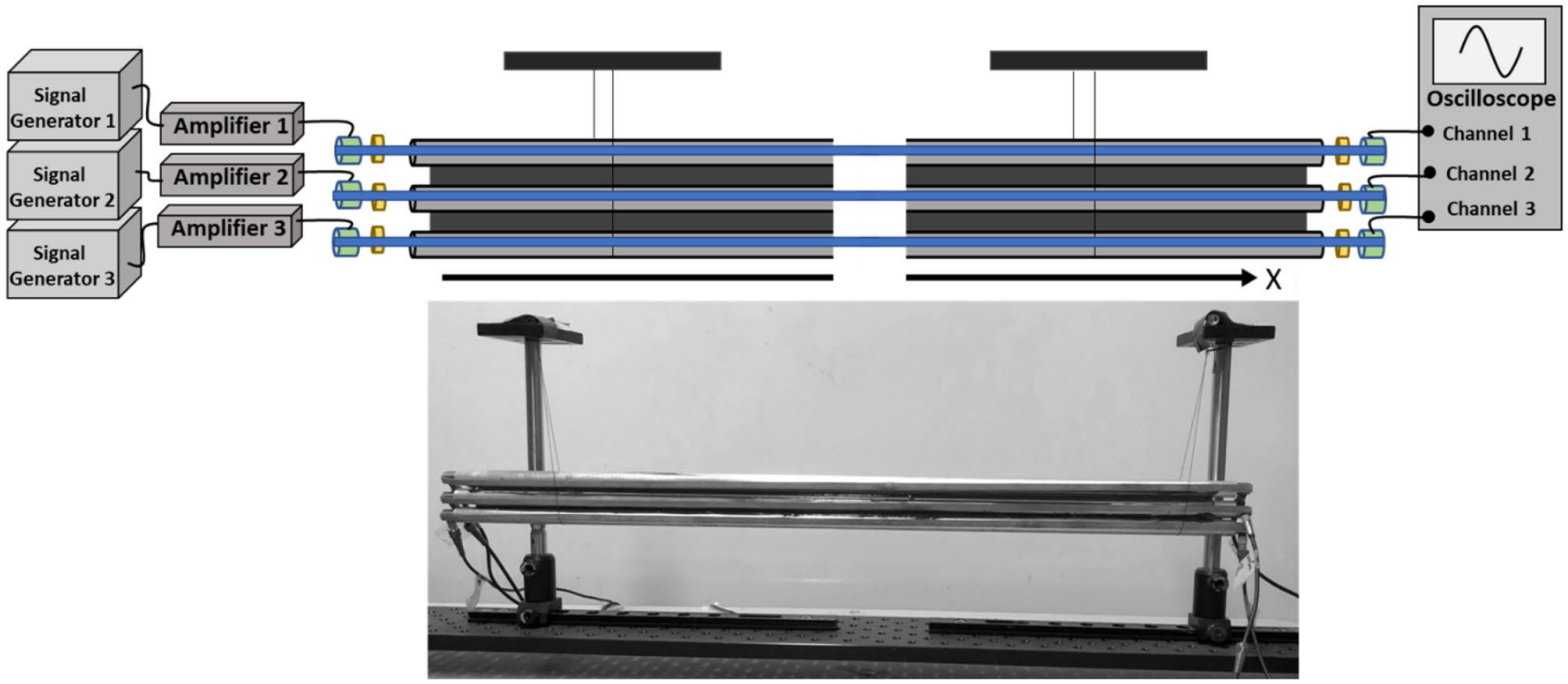
Picture and exploded-view of an array of three acoustic waveguides coupled with epoxy resin and schematic illustration of the experimental system for generating and detecting logical phi-bits. Three separate signal generators and amplifiers are used to drive piezoelectric transducers. Driving and detecting transducers are attached to the opposite ends of the rod-like acoustic waveguides by the pressure of three independent rubber bands. A thin layer of honey is used as ultrasonic coupling agent between the transducers and the rod ends. The signals generated by the detecting transducers enter an oscilloscope via independent input channels for analysis. The array of waveguides is suspended by thin threads for isolation.

We have employed a metamaterial composed of coupled elastic wave guides to physically realize acoustic phi-bits^3^. The waveguides consist of three aluminum rods (6061 aluminum with diameter 1.27 cm, length 0.609 m, and density 2660 $\mathrm{kg}/{\mathrm{m}}^{3}$). When driven externally at two primary frequencies, an array of three elastically coupled acoustic waveguides can produce a displacement field, which when partitioned in the frequency domain leads to modes with secondary frequencies associated with logical phi-bits^3^. The waveguides are arranged in a linear array with epoxy-filled lateral gaps (50176 KwikWeld Syringe) (see Fig. 1).

Ultrasonic longitudinal contact transducers (V133-RM, Olympus IMS) drive and detect the acoustic field at the rod ends. The transducers are coupled to the aluminum using commercially available honey. Separate waveform generators (B&K Precision 4055B) through PD200 amplifiers (high bandwidth, low-noise linear amplifier) excite two driving transducers at the ends of two waveguides with sinusoidal signals at frequency ${f}_{1}$ and ${f}_{2}$. Three detecting transducers at the opposite ends collect data on the displacement field. Each detecting transducers connect to a separate channel of a Tektronix oscilloscope (MDO3024) to record signals and provide temporal and spatial information across the array.

By wrapping rubber bands (supersize bands from Walmart, 564755837) around the transducer/rod assembly and employing honey as ultrasonic coupling agent between the transducers and the rods we optimize the resolution of the acoustic modes. The rubber bands maintain a uniform pressure on the transducers throughout all experiments.

Temporal Fourier transforms of output signals generate the spectral information. The displacement field measured at the waveguide’s detection-ends are the Fourier sum of modes with the primary frequencies ${f}_{1}$ and ${f}_{2}$ as well as secondary modes which frequencies are linear combination of the driving frequencies: $p{f}_{1}+q{f}_{2}$. In^3^ we defined a logical phi-bit as a two-level, secondary mode of vibration whose state is characterized by the set of frequency coefficients $\{p,q\}$ and spatial mode associated with two independent relative phases of the displacement between the waveguides. For an externally driven three-waveguide system, we define the state of a phi-bit, “*j*”, by a $2\times 1$ vector: $${\overrightarrow{U}}_{(j)}=\left(\begin{array}{c}{\widehat{c}}_{2}{e}^{i{\varphi }_{12}^{(j)}}\\ {\widehat{c}}_{3}{e}^{i{\varphi }_{13}^{(j)}}\end{array}\right){e}^{i{\omega }^{(j)}t}$$ where the angular frequency ${\omega }^{(j)}$ is the linear combination of the driving angular frequencies with coefficients $\{p,q\}$. The magnitudes ${\widehat{C}}_{2}$ and ${\widehat{C}}_{3}$ are normalized to that of the first waveguide, and ${\varphi }_{12}^{(j)}={\varphi }_{2}^{(j)}-{\varphi }_{1}^{(j)}$ and ${\varphi }_{13}^{(j)}={\varphi }_{3}^{(j)}-{\varphi }_{1}^{(j)}$ are the two independent phases in waveguides 2 and 3 relative to waveguide 1. The amplitude and phases at the waveguide ends can be measured unambiguously. A single phi-bit state lives in a 2D Hilbert space ${h}_{(j)}$ and spans the Bloch sphere.

The phi-bits co-locate within the same physical space and are subject to distance-independent interactions. Tensor-product structures of systems that comprise many logical phi-bits (*P*) can support non-separable states in scalable, exponentially complex Hilbert spaces. Indeed, a noninteracting *P* phi-bit system’s state is the tensor products of single phi-bit states, namely: $\overrightarrow{W}={\overrightarrow{U}}_{(1)}\otimes \dots \otimes {\overrightarrow{U}}_{(P)}$. The tensor product of the basis vectors of single phi-bits forms a complete basis for the states of the noninteracting multi phi-bit system. This basis defines a ${2}^{P}$ dimensional Hilbert space *H*, which is the tensor product of the *P* Hilbert spaces of the individual noninteracting phi-bits, $H={h}_{(1)}\otimes \dots \otimes {h}_{(P)}$. The secondary modes (i.e., the phi-bits) correlate via the possible interactions of the waveguide-transducer-amplifier-generator assembly. For correlated phi-bits, the Hilbert space is the same as for a noninteracting system; however, a state of the interacting system may be a separable or non-separable linear combination (with complex coefficients) of the basis vectors of *H*. The state of correlated phi-bits within *H* can be manipulated^4^ to achieve quantum-like unitary operation or even algorithms that are analogous to quantum gates used in quantum computing^5,6^.

The logical phi-bits and their correlation are the result of nonlinear phenomena within the physical system of Fig. 1. Recent developments in the use of nonlinear ultrasonic techniques for non-destructive testing (NDT) of materials exploiting subharmonic and harmonic waves and guided waves^7–9^ provide guidance as to what might be the factors leading to the nonlinearity in our system^10^. The support conditions of the array of waveguides may lead to nonlinearities through support/array contact. We have used suspension of the array of waveguides by two threads as it is known to minimize the possibility of support/sample nonlinearities in ultrasonic NDT^10^. It is well known that amplifiers, pre-amplifiers and signal generators exhibit so called “harmonic distortion.” That is, these electronic systems designed to generate one single desired frequency, *f*, also produce its harmonics 2*f*, 3*f*, etc. Indeed, we have verified that our signal generators/amplifiers exhibit some level of harmonic distortion. We therefore do not consider secondary modes that are simple harmonics of the driving frequencies ${f}_{1}$ or ${f}_{2}$ to be reliable candidate for serving as phi-bits. Secondary mode with frequency $p{f}_{1}+q{f}_{2}$ may result from “intermodulation distortion”. However, this commonly arises from feeding two signals with different frequencies ${f}_{1}$ and ${f}_{2}$ into a single nonlinear electronic system (signal generator and amplifiers) that will then produces the multiple frequencies ${af}_{1}+b{f}_{2}$ where $a, b$ are integers^10^. In our system, we have separated the excitations by using separate signal generator/amplifier for each applied frequency, ${f}_{1}$ or ${f}_{2}$. In this paper, the bottom rod of the waveguide assembly is excited at ${f}_{1}$ and the middle rod is excited at ${f}_{2}.$ Separating the two excitations physically and electronically ensures that the nonlinear mixing of the frequencies occurs in the array of waveguides. However, the approach using separate amplifiers and transducers has been shown to not eliminate but only reduce the mixing of signals^10^. On the detection side, the signals generated by the three detecting transducers are acquired through three independent input channels on the oscilloscope.

Although piezoelectric materials are intrinsically nonlinear, we operate the ultrasonic transducers with amplifiers. Furthermore, we employ three individual transducers at the driving ends of the array of three waveguides to minimize the potential for frequency mixing. Fluid and gel ultrasonic coupling agents can introduce nonlinearity^10^. By applying a constant pressure at the transducer/honey/rod end interface using stretched rubber bands, we minimize the thickness of the coupling agent and reduce its potential contribution to nonlinear phenomena.

The restoring force of a stretched rubber band is intrinsically nonlinear^11^. The three rubber bands utilized to hold the driving and detecting transducers at the ends of each rod may therefore possibly contribute to the nonlinearity of the system. Finally, experimental measurements of the compression-tension asymmetry in the modulus of epoxy resins under various strain rates indicates that the epoxy used to couple the waveguides in our system may also contribute to the nonlinearities of the system^12^.

## Experimental results

**Figure 2:**
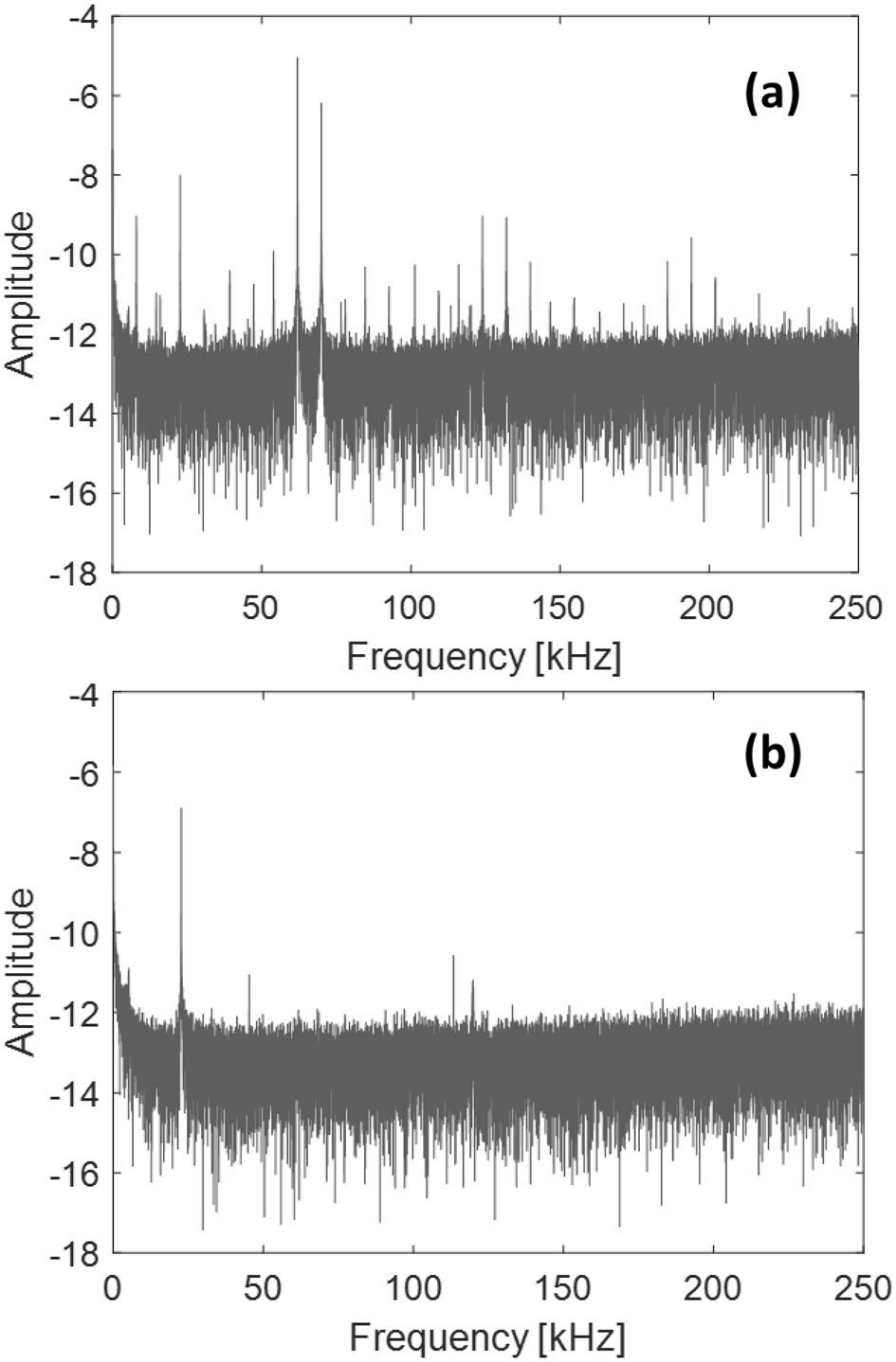
Fourier frequency Spectrum calculated from velocity time series measured using a laser Doppler vibrometer on third rod of the array of waveguides (**a**) and on the corresponding rubber band (**b**). The amplitudes are in dB. The 22 kHz peak in (**b**) is an artifact from the laser Doppler vibrometer.

First, we conducted an experiment to shed light on the contribution of the rubber bands to the nonlinear behavior of the array of waveguides. We drive the system with the following frequencies: ${f}_{1}=62 \, {\text{kHz}}$ and ${f}_{2}=66\, {\text{kHz}}$. At these frequencies the wavelength of longitudinal waves in aluminum is on the order of 10 cm making propagation along the rod-like waveguides nearly one dimensional^1^. Furthermore, at these frequencies the linear longitudinal modes of the finite length waveguides are well defined and the transducers give satisfactory driving and detecting amplitudes^1^. We use a noncontact full-field scanning laser Doppler vibrometer (Polytec PSV-400) to measure the velocity field in the third waveguide as well as in the rubber band. The PSV-400 laser head was connected to a controller (Polytec OFV-5000) utilizing the VD-07 velocity decoder, which is sensitive to velocities in the range of 10 mm/s up to frequencies of MHz. Since the vibrometer can only measure velocities that are parallel to its beam, and to not rely small contraction/expansion due to Poisson’s ratio perpendicular to the longitudinal direction, the laser head was placed at an oblique angle to the side of the rods to measure the longitudinal vibrations, without compromising its signal strength. Retroreflective tape was attached on the surface of the rod and rubber band to optimize the signal. The velocity time series at one point along the rod and one point along a rubber band are Fourier transformed and their corresponding frequency spectra are reported in Fig. 2. The frequency spectrum of the rod clearly shows the primary driving modes but also the presence of nonlinear modes. The modes with frequency $p{f}_{1}+q{f}_{2}$ are phi-bit modes. The values of $p$ and $q$ are determined by the order of nonlinearity present in the system. Figure 2 shows that the current array of nonlinear waveguides enables the realization of a large number of combinations of values for $p$ and $q$. The frequency spectrum of the rubber band shows little to no contribution to the nonlinear modes of vibration of the system. We can therefore neglect the contribution from the rubber bands to the nonlinearity of the system. All subsequent measurements of phi-bit characteristics are performed using transducers at the detection ends of the array of waveguides.

**Figure 3:**
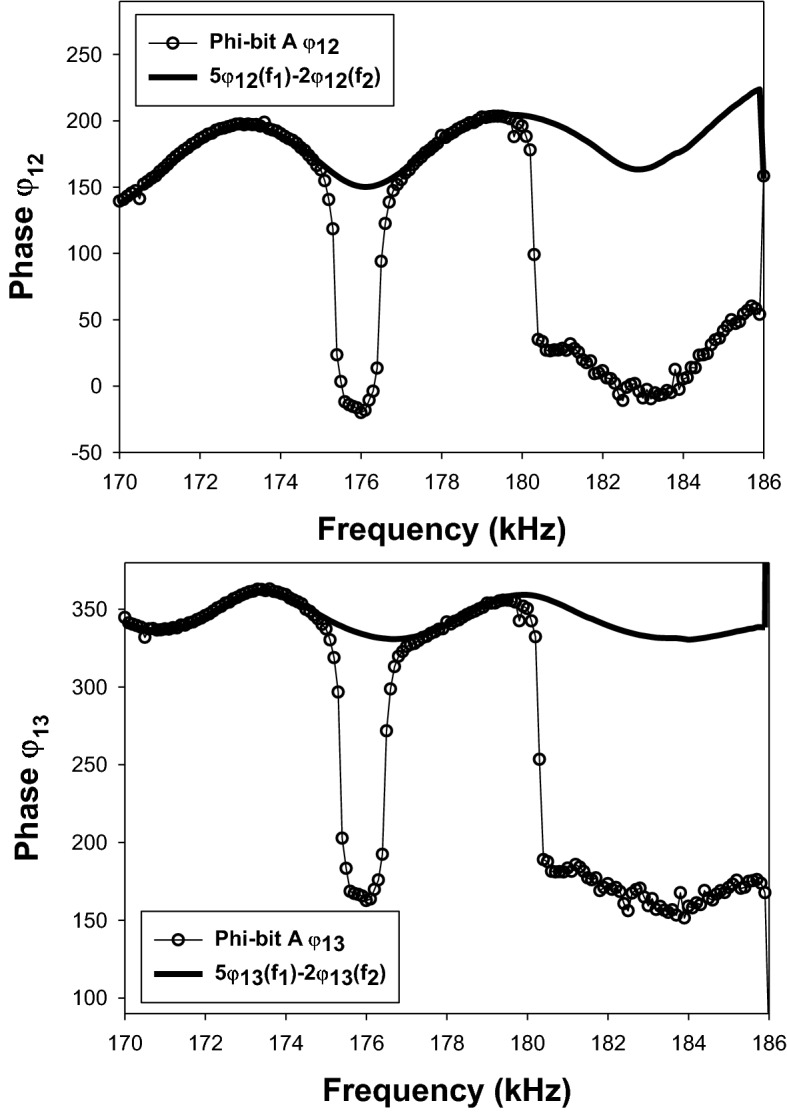
Phases ${\varphi }_{12}$ and ${\varphi }_{13}$ (open circles) measured for the phi-bit “A” defined by the mixed frequency $5{f}_{1}-2{f}_{2}$ when ${f}_{1}=62\, {\text{kHz}}$ and the second driving frequency is varied in the interval [70–62 kHz] by decreasing increments of 50 Hz. In both graphs the thick solid line corresponds to the linear combination of phases $5{\varphi }_{12 }\left({f}_{1}\right)-2{\varphi }_{12 }\left({f}_{2}\right)$ and $5{\varphi }_{13 }\left({f}_{1}\right)-2{\varphi }_{13 }\left({f}_{2}\right)$ where ${\varphi }_{12 }\left({f}_{\mathrm{1,2}}\right)$ and ${\varphi }_{13 }\left({f}_{\mathrm{1,2}}\right)$ are the phases of the primary modes at the driving frequencies.

**Figure 4:**
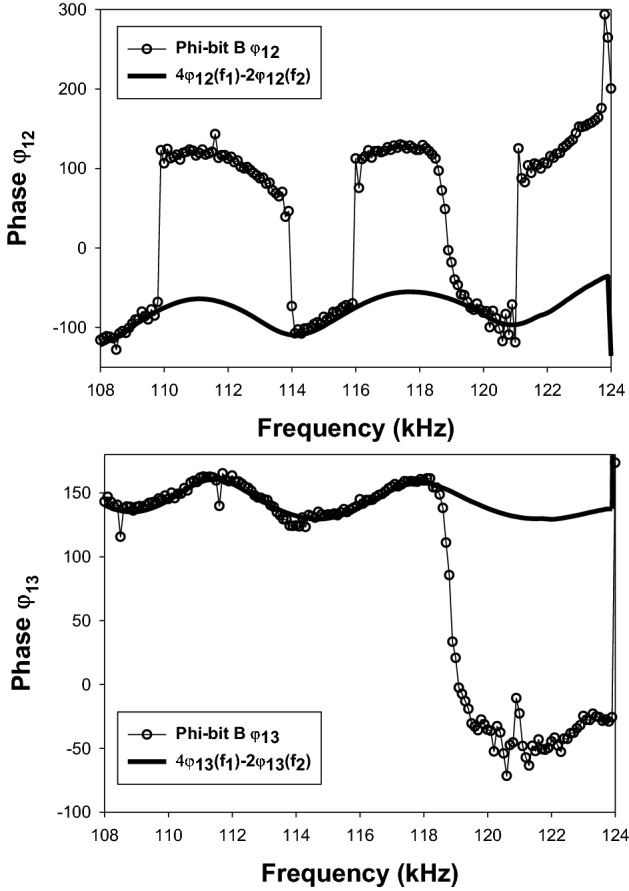
Phases ${\varphi }_{12}$ and ${\varphi }_{13}$ (open circles) measured for the phi-bit “B” defined by the mixed frequency $4{f}_{1}-2{f}_{2}$ when ${f}_{1}=62 \, {\text{kHz}}$ and the second driving frequency is varied in the interval [70–62 kHz] by decreasing increments of 50 Hz. In both graphs the thick solid line corresponds to the linear combination of phases $4{\varphi }_{12 }\left({f}_{1}\right)-2{\varphi }_{12 }\left({f}_{2}\right)$ and $4{\varphi }_{13 }\left({f}_{1}\right)-2{\varphi }_{13 }\left({f}_{2}\right)$ where ${\varphi }_{12 }\left({f}_{\mathrm{1,2}}\right)$ and ${\varphi }_{13 }\left({f}_{\mathrm{1,2}}\right)$ are the phases of the primary modes at the driving frequencies.

Figure 3 illustrates the behavior of the experimentally measured phases ${\varphi }_{12}$ and ${\varphi }_{13}$ for the phi-bit defined by the mixed frequency $5{f}_{1}-2{f}_{2}$ when ${f}_{1}=62\, {\text{kHz}}$ and the second driving frequency is varied in the interval [70–62 kHz] by increments of 50 Hz. This phi-bit is subsequently labelled “phi-bit A.” In addition, we have measured the phases ${\varphi }_{12 }\left({f}_{\mathrm{1,2}}\right)$ and ${\varphi }_{13 }\left({f}_{\mathrm{1,2}}\right)$ for the primary modes observed at the frequencies ${f}_{1}$ and ${f}_{2}$. Since ${f}_{1}$ does not change in the experiment, the associated phases remain constant. However, the frequency ${f}_{2}$ varying, the corresponding phases vary. We have calculated the quantities ${\varphi }_{12}^{b}(A)=5{\varphi }_{12 }\left({f}_{1}\right)-2{\varphi }_{12 }\left({f}_{2}\right)$ and ${\varphi }_{13}^{b}(A){=5\varphi }_{13 }\left({f}_{1}\right)-2{\varphi }_{13 }\left({f}_{2}\right)$ which are also plotted in Fig. 3. The phases ${\varphi }_{12}(A)$ and ${\varphi }_{13}(A)$ exhibit several remarkable features in the form of upward or downward jumps superposed onto the background functions ${\varphi }_{12}^{b}(A)$ and ${\varphi }_{13}^{b}(A)$. The jumps amount to phase changes on the order of π (180°). In the case of phi-bit A, there are the same number of π jumps for ${\varphi }_{12}$ and ${\varphi }_{13}$ over the range of frequency studied and they occur at the same frequencies. The jumps are very sharp and happen over ranges of frequency on the order of 100 to 200 Hz. In contrast, the calculated background phases oscillate with periods on the order of several thousands of Hz. However, other logical phi-bits exhibit different behaviors. For instance, Fig. 4 shows that the behavior of the measured phases ${\varphi }_{12}$ and ${\varphi }_{13}$ for the phi-bit defined by the mixed frequency $5{f}_{1}-2{f}_{2}$ (labelled phi-bit “B”) do not parallel each other. Here, ${\varphi }_{12}$ undergoes five jumps while ${\varphi }_{13}$ shows only one. These jumps occur over very narrow ranges of frequency not exceeding a few hundred Hz. However, similarly to phi-bit A, phi-bit B is also the superposition of jumps onto background phases ${\varphi }_{12}^{b}(B)=4\left({f}_{1}\right)-2{\varphi }_{12 }\left({f}_{2}\right)$ and ${\varphi }_{13}^{b}(B){=4\varphi }_{13 }\left({f}_{1}\right)-2{\varphi }_{13 }\left({f}_{2}\right)$. We have also observed (not shown here) phi-bits that do not show any π jump in either ${\varphi }_{12}$ and ${\varphi }_{13}$ or phi-bits that exhibit jumps in ${\varphi }_{12}$ but not in ${\varphi }_{13}$ and vise versa. In all cases, for any phi-bit $p{f}_{1}+q{f}_{2}$, showing π jumps or not, the phases ${\varphi }_{12}$ and ${\varphi }_{13}$ superpose onto the backgrounds $p{\varphi }_{12 }\left({f}_{1}\right)+q{\varphi }_{12 }\left({f}_{2}\right)$ and $p{\varphi }_{13 }\left({f}_{1}\right)+q{\varphi }_{13 }\left({f}_{2}\right)$ for all frequency ranges studied. For all phi-bits, the background phases show oscillations as functions of frequency of several thousand Hz while the π jumps occur over much shorter frequency intervals of at most a few hundred Hz.

**Figure 5:**
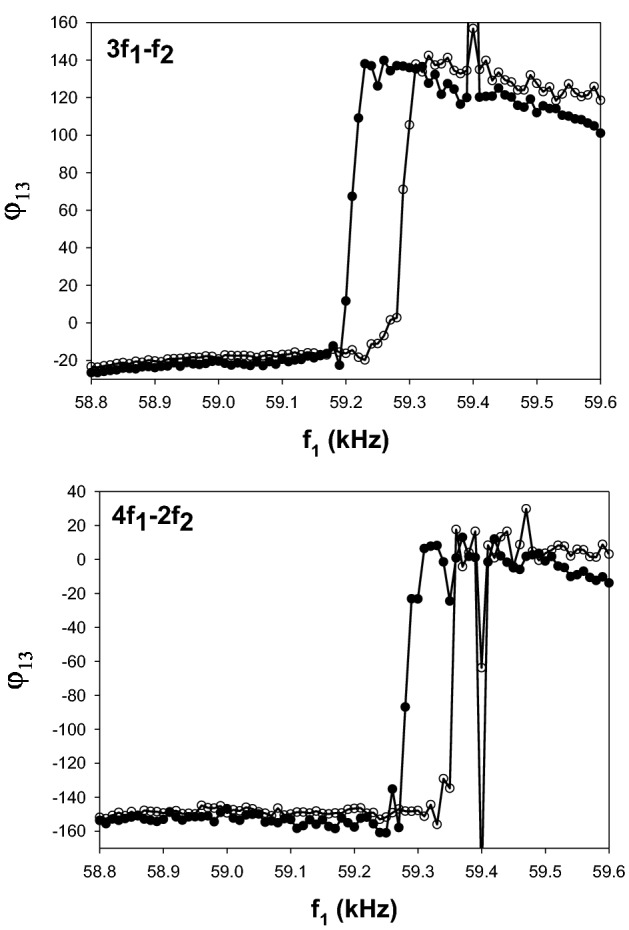
Phase ${\varphi }_{13}$ for two phi-bits $3{f}_{1}-1{f}_{2}$ (top) and $4{f}_{1}-2{f}_{2}$ (bottom) as a function of frequency ${f}_{1}$ tuned downward or upward. The closed circles correspond to the conditions when ${f}_{1}$ is decreased by increments of 10 Hz from $59.6 \, {\text{kHz}}$ to $58.8 \, {\text{kHz}}$ and the second driving frequency is fixed at ${f}_{2}=66 \, {\text{kHz}}$. The open circles correspond to ${f}_{1}$ increasing by increments of 10 Hz from $58.8 \, {\text{kHz}}$ to $59.6 \, {\text{kHz}}$ and the second driving frequency remaining fixed at ${f}_{2}=66 \, {\text{kHz}}$. The origin of the spikes near 59.4 kHz is unknown.

We investigated the jumps further by conducting experiments whereas the frequency ${f}_{2}$ is fixed at $66 \, {\text{kHz}}$ and ${f}_{1}$ is decreased by increments of 10 Hz from $59.6 \, {\text{kHz}}$ to $58.8 \, {\text{kHz}}$ or ${f}_{1}$ is increased by increments of 10 Hz from $58.8 \, {\text{kHz}}$ to $59.6 \, {\text{kHz}}.$ Figure 5 shows the difference in phase ${\varphi }_{13}$ for two phi-bits, namely phi-bit B and a phi-bit C with $3{f}_{1}-1{f}_{2}$. With a fine 10 Hz detuning, we note that the phase jumps occur over a narrow range of frequency ranging from 40 to 100 Hz. We also observe a hysteretic behavior as the frequency increases or decreases. The width of the hysteresis is less than 100 Hz. We also note that with the finer tuning interval the phase jumps are actually less than π. These jumps are closer to 160 than 180 degrees. It is worth noting that some other phi-bits we have investigated do not exhibit hysteretic phase jumps. As will be seen in Section “Possible origin of the π jumps”, hysteretic behavior results from nonlinear instability which may not be present in some nonlinear modes.

We have tested the system when the frequency ${f}_{1}$ is fixed at $62 \, {\text{kHz}}$ and ${f}_{2}$ is decreased by increments of 50 Hz from $78 \, {\text{kHz}}$ to $70 \, {\text{kHz}}$ or ${f}_{2}$ is increased by increments of 50 Hz from $70 \, {\text{kHz}}$ to $78 \, {\text{kHz}}.$ These experimental results (not reported here) show hysteretic phase jumps similar to those in Fig. 5. These confirm the general presence of hysteresis loops for various sets of frequencies.

These experimental results broach the challenging questions of the origin of the experimentally observed background and jumps in the phases ${\varphi }_{12}$ and ${\varphi }_{13}$, this in the context of the sources of nonlinearity of the system. The subsequent sections develop perturbative models of nonlinear driven array of acoustic waveguides to offer possible answers to these questions.

## Possible origin of the phase backgrounds

**Figure 6:**
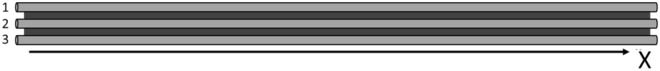
Schematic of the metamaterial composed of a parallel array of three coupled waveguides.

We consider a model of the acoustic metamaterial composed of three one-dimensional elastic waveguides coupled elastically along their length (Fig. 6). Each waveguide is driven externally at its end at the position $x=0$. $x$ represents the position along the waveguides.

The nonlinear elastic wave equation in the long wavelength limit is written as:

1 $$\left[\left(\frac{{\partial }^{2}}{{\partial t}^{2}}-{\beta }^{2}\frac{{\partial }^{2}}{{\partial x}^{2}}+\mu \frac{\partial }{\partial t}\right)\overleftrightarrow{I}+{\alpha }^{2}\overleftrightarrow{M}\right]\overset{\lower0.5em\hbox{$\smash{\scriptscriptstyle\rightharpoonup}$}}{U}+\varepsilon \overset{\lower0.5em\hbox{$\smash{\scriptscriptstyle\rightharpoonup}$}}{G}(\overset{\lower0.5em\hbox{$\smash{\scriptscriptstyle\rightharpoonup}$}}{U})={\overset{\lower0.5em\hbox{$\smash{\scriptscriptstyle\rightharpoonup}$}}{F}}_{1}{\delta }_{x=0}\mathrm{cos}{\omega }_{1}t+{\overset{\lower0.5em\hbox{$\smash{\scriptscriptstyle\rightharpoonup}$}}{F}}_{2}{\delta }_{x=0}\mathrm{cos}{\omega }_{2}t$$

The parameter *β* is proportional to the speed of sound along the waveguides. The parameter $\mu$ represents damping. $\overleftrightarrow{I}$ is the identity matrix. $\alpha$ measures the elastic coupling strength between waveguides due to the epoxy. $\overleftrightarrow{M}$ is the matrix characterizing the elastic coupling between the three waveguides. In the case of our planar array of waveguides, the coupling matrix takes the form:

2 $$\overleftrightarrow{M}=\left(\begin{array}{ccc}1& -1& 0\\ -1& 2& -1\\ 0& -1& 1\end{array}\right)$$

$${\overset{\lower0.5em\hbox{$\smash{\scriptscriptstyle\rightharpoonup}$}}{F}}_{1}$$ and $${\overset{\lower0.5em\hbox{$\smash{\scriptscriptstyle\rightharpoonup}$}}{F}}_{2}$$ are 3 $$\times 1$$ vectors representing the external driving harmonic forces for the two different driving angular frequencies $${\omega }_{1}=2\pi {f}_{1}$$ and $${\omega }_{2}=2\pi {f}_{2}$$.

The displacement in waveguides 1, 2 and 3 is represented by the $$3\times 1$$ vector $$\overset{\lower0.5em\hbox{$\smash{\scriptscriptstyle\rightharpoonup}$}}{U}=\left({U}_{1},{U}_{2},{U}_{3}\right)$$. $$\varepsilon \overset{\lower0.5em\hbox{$\smash{\scriptscriptstyle\rightharpoonup}$}}{G}(\overset{\lower0.5em\hbox{$\smash{\scriptscriptstyle\rightharpoonup}$}}{U})$$ is a nonlinear term with strength $$\varepsilon$$. The effect of this nonlinear term will be addressed in Section “Possible origin of the π jumps”. Here, we set $$\varepsilon$$=0 and solve the linear wave equation:

3 $$\left[\left(\frac{{\partial }^{2}}{{\partial t}^{2}}-{\beta }^{2}\frac{{\partial }^{2}}{{\partial x}^{2}}+\mu \frac{\partial }{\partial t}\right)\overleftrightarrow{I}+{\alpha }^{2}\overleftrightarrow{M}\right]{\overset{\lower0.5em\hbox{$\smash{\scriptscriptstyle\rightharpoonup}$}}{U}}^{(0)}={\overset{\lower0.5em\hbox{$\smash{\scriptscriptstyle\rightharpoonup}$}}{F}}_{1}{\delta }_{x=0}{e}^{i{\omega }_{1}t}+{\overset{\lower0.5em\hbox{$\smash{\scriptscriptstyle\rightharpoonup}$}}{F}}_{2}{\delta }_{x=0}{e}^{i{\omega }_{2}t}$$

$${\overset{\lower0.5em\hbox{$\smash{\scriptscriptstyle\rightharpoonup}$}}{U}}^{(0)}$$ is the linear solution. In Eq. (3), we have replaced the trigonometric functions associated with the drivers by complex exponentials.

We can solve this equation by defining by $${\lambda }_{n}$$ and $${\overset{\lower0.5em\hbox{$\smash{\scriptscriptstyle\rightharpoonup}$}}{E}}_{n}$$ with *n* = 1, 2, 3, the eigen values and eigen vectors of the $$\overleftrightarrow{M}$$ matrix, where $${\overset{\lower0.5em\hbox{$\smash{\scriptscriptstyle\rightharpoonup}$}}{E}}_{n}$$ represent the spatial eigen modes across the waveguides with components $${E}_{n,j}$$, $$j=\mathrm{1,2},3$$. We write:

4 $$\overleftrightarrow{M}{\overset{\lower0.5em\hbox{$\smash{\scriptscriptstyle\rightharpoonup}$}}{E}}_{n}={\lambda }_{n}{\overleftrightarrow{I} \overset{\lower0.5em\hbox{$\smash{\scriptscriptstyle\rightharpoonup}$}}{E}}_{n}$$

The eigen modes have eigen values ${\lambda }_{1}=0{, \lambda }_{2}=1$, and ${\lambda }_{3}=3$, and are given by:

$${\overset{\lower0.5em\hbox{$\smash{\scriptscriptstyle\rightharpoonup}$}}{E}}_{1}=\left(\begin{array}{c}{E}_{\mathrm{1,1}}\\ {E}_{\mathrm{1,2}}\\ {E}_{\mathrm{1,3}}\end{array}\right)=\frac{1}{\sqrt{3}}\left(\begin{array}{c}1\\ 1\\ 1\end{array}\right),{\overset{\lower0.5em\hbox{$\smash{\scriptscriptstyle\rightharpoonup}$}}{E}}_{2}=\left(\begin{array}{c}{E}_{\mathrm{2,1}}\\ {E}_{\mathrm{2,2}}\\ {E}_{\mathrm{2,3}}\end{array}\right)=\frac{1}{\sqrt{2}}\left(\begin{array}{c}1\\ 0\\ -1\end{array}\right), {\overset{\lower0.5em\hbox{$\smash{\scriptscriptstyle\rightharpoonup}$}}{E}}_{3}=\left(\begin{array}{c}{E}_{\mathrm{3,1}}\\ {E}_{\mathrm{3,2}}\\ {E}_{\mathrm{3,3}}\end{array}\right)=\frac{1}{\sqrt{6}}\left(\begin{array}{c}1\\ -2\\ 1\end{array}\right)$$

We can now expand the displacement vector on the complete orthonormal basis, $$\left\{{\overset{\lower0.5em\hbox{$\smash{\scriptscriptstyle\rightharpoonup}$}}{E}}_{n}\right\}$$:

5 $${\overset{\lower0.5em\hbox{$\smash{\scriptscriptstyle\rightharpoonup}$}}{U}}^{(0)}=\sum_{n}{u}_{n}^{(0)}{\overset{\lower0.5em\hbox{$\smash{\scriptscriptstyle\rightharpoonup}$}}{E}}_{n}$$

Since Eq. (3) is linear, we focus on an external force with a single driving frequency, $${\overset{\lower0.5em\hbox{$\smash{\scriptscriptstyle\rightharpoonup}$}}{F}}_{l}$$ with *l* = 1 or 2. We seek solutions of the simplified equation:

6 $$\left[\left(\frac{{\partial }^{2}}{{\partial t}^{2}}-{\beta }^{2}\frac{{\partial }^{2}}{{\partial x}^{2}}+\mu \frac{\partial }{\partial t}\right)\overleftrightarrow{I}+{\alpha }^{2}\overleftrightarrow{M}\right]{\overset{\lower0.5em\hbox{$\smash{\scriptscriptstyle\rightharpoonup}$}}{U}}_{l}^{(0)}={\overset{\lower0.5em\hbox{$\smash{\scriptscriptstyle\rightharpoonup}$}}{F}}_{l}{\delta }_{x=0}{e}^{i{\omega }_{l}t}$$

The $$3\times 1$$ vector, $${\overset{\lower0.5em\hbox{$\smash{\scriptscriptstyle\rightharpoonup}$}}{F}}_{l}$$, is also expanded on the basis $$\left\{{\overset{\lower0.5em\hbox{$\smash{\scriptscriptstyle\rightharpoonup}$}}{E}}_{n}\right\}$$ :

7 $${\overset{\lower0.5em\hbox{$\smash{\scriptscriptstyle\rightharpoonup}$}}{F}}_{l}=\sum_{n}{F}_{n}^{(l)}{\overset{\lower0.5em\hbox{$\smash{\scriptscriptstyle\rightharpoonup}$}}{E}}_{n}$$

Inserting Eqs. (4), (5) and (7) into Eq. (6) yields a set of 3 equations of the form:

8 $$\left(\frac{{\partial }^{2}}{{\partial t}^{2}}-{\beta }^{2}\frac{{\partial }^{2}}{{\partial x}^{2}}+\mu \frac{\partial }{\partial t}+{\alpha }^{2}{\lambda }_{n}\right){u}_{n,l}^{(0)}={F}_{n}^{(l)}{\delta }_{x=0}{e}^{i{\omega }_{l}t}$$

The coefficients ${u}_{n,l}^{(0)}$ are now expanded on plane waves which follow the harmonic driving force:

9 $${u}_{n,l}^{(0)}=\sum_{{k}_{n}}{A}_{n,l}({k}_{n}){e}^{i{k}_{n}x}{e}^{i{\omega }_{l}t}$$

Since the waveguides are finite in length, the wave numbers, ${k}_{n}$, form a discrete set and we use a discrete summation in Eq. (9).

Equation (8) is now evaluated at $x=0$ leading to the particular solutions for the driven complex amplitudes:

10 $${A}_{n,l}\left({k}_{n}\right)=\frac{{F}_{n}^{(l)}}{{\omega }_{0,n}^{2}\left({k}_{n}\right)-{\omega }_{l}^{2}+i\mu {\omega }_{l}}$$

where we define the characteristic frequency

11 $${\omega }_{0,n}^{2}\left({k}_{n}\right)={\beta }^{2}{k}_{n}^{2}+{\alpha }^{2}{\lambda }_{n}$$

The particular linear displacement field is found to be

12 $${\overset{\lower0.5em\hbox{$\smash{\scriptscriptstyle\rightharpoonup}$}}{U}}_{l}^{(0)}=\sum_{n=1}^{3}{\overset{\lower0.5em\hbox{$\smash{\scriptscriptstyle\rightharpoonup}$}}{E}}_{n}\sum_{{k}_{n}}{A}_{n,l}({k}_{n}){e}^{i{k}_{n}x}{e}^{i{\omega }_{l}t}$$

with the complex resonant amplitudes given by Eq. (10). We note that these amplitudes are complex quantities as a result of the dissipative term $i\mu \omega$ and therefore possess a phase. Rewriting the linear displacement field for driving forces with two different frequencies, one gets:

13 $${\overset{\lower0.5em\hbox{$\smash{\scriptscriptstyle\rightharpoonup}$}}{U}}^{(0)}={\overset{\lower0.5em\hbox{$\smash{\scriptscriptstyle\rightharpoonup}$}}{U}}_{1}^{(0)}+{\overset{\lower0.5em\hbox{$\smash{\scriptscriptstyle\rightharpoonup}$}}{U}}_{2}^{(0)}=\sum_{n=1}^{3}{\overset{\lower0.5em\hbox{$\smash{\scriptscriptstyle\rightharpoonup}$}}{E}}_{n}\left(\sum_{{k}_{n}}{A}_{n,1}({k}_{n}){e}^{i{k}_{n}x}{e}^{i{\omega }_{1}t}+\sum_{{k}_{n}^{^{\prime}}}{A}_{n,2}({k}_{n}^{^{\prime}}){e}^{i{k}_{n}^{^{\prime}}x}{e}^{i{\omega }_{2}t}\right)$$

Here, we have used two independent summation indices for the wavenumber, namely ${k}_{n}$ and ${k}_{n}^{^{\prime}}$.

We can rewrite the contribution of the two driving frequencies to Eq. (13) by using

14 $${\overset{\lower0.5em\hbox{$\smash{\scriptscriptstyle\rightharpoonup}$}}{U}}_{1}^{(0)}=\left(\begin{array}{c}{C}_{1}^{(1)}{e}^{i{\varphi }_{1}^{(1)}}\\ {C}_{2}^{(1)}{e}^{i{\varphi }_{2}^{(1)}}\\ {C}_{3}^{(1)}{e}^{i{\varphi }_{3}^{(1)}}\end{array}\right){e}^{i{\omega }_{1}t}\mathrm{ and }{\overset{\lower0.5em\hbox{$\smash{\scriptscriptstyle\rightharpoonup}$}}{U}}_{2}^{(0)}=\left(\begin{array}{c}{C}_{1}^{(2)}{e}^{i{\varphi }_{1}^{(2)}}\\ {C}_{2}^{(2)}{e}^{i{\varphi }_{2}^{(2)}}\\ {C}_{3}^{(2)}{e}^{i{\varphi }_{3}^{(2)}}\end{array}\right){e}^{i{\omega }_{2}t}$$

We can also define the displacement field at the end of the waveguides due to the coupled vibration by the renormalized $2\times 1$ vectors:

$${\overset{\lower0.5em\hbox{$\smash{\scriptscriptstyle\rightharpoonup}$}}{U}}_{1}^{(0)}={\left(\begin{array}{c}1\\ {\widehat{C}}_{2}{e}^{i{\varphi }_{12}}\\ {\widehat{C}}_{3}{e}^{i{\varphi }_{13}}\end{array}\right)}^{(1)}{e}^{i{\omega }_{1}t} \mathrm{and }{\overset{\lower0.5em\hbox{$\smash{\scriptscriptstyle\rightharpoonup}$}}{U}}_{2}^{(0)}={\left(\begin{array}{c}1\\ {\widehat{C}}_{2}{e}^{i{\varphi }_{12}}\\ {\widehat{C}}_{3}{e}^{i{\varphi }_{13}}\end{array}\right)}^{(2)}{e}^{i{\omega }_{2}t}$$

where ${\widehat{C}}_{2}$ and ${\widehat{C}}_{3}$ are normalized to ${C}_{1}$ and ${\varphi }_{12}={\varphi }_{2}-{\varphi }_{1}$ and ${\varphi }_{13}={\varphi }_{3}-{\varphi }_{1}$. Here ${\varphi }_{12}^{(1)}$, ${\varphi }_{13}^{(1)}$, ${\varphi }_{12}^{(2)}$,and ${\varphi }_{13}^{(2)}$ correspond to the experimentally measure phases ${\varphi }_{12 }\left({f}_{1}\right)$, ${\varphi }_{13 }\left({f}_{1}\right)$, ${\varphi }_{12 }\left({f}_{2}\right)$, and ${\varphi }_{13 }\left({f}_{1}\right)$ of the primary modes. These are the phases that determine the calculated phase backgrounds discussed in the experimental section. So far, our model focuses on the array of waveguides. However, in accordance with Section “Physical system, logical phi-bits and sources of nonlinearity”, we propose a model of the array of waveguides coupled to extrinsic nonlinear damped oscillators at the rod ends that may be representative of nonlinear effects associated with the amplifiers and/or transducers and/or coupling agent:

15 $$\left(\frac{{d}^{2}}{{dt}^{2}}+{\omega }_{R}^{2}+{\mu }_{R}\frac{d}{dt}\right)\overrightarrow{V}+{\varepsilon }_{R}\overset{\lower0.5em\hbox{$\smash{\scriptscriptstyle\rightharpoonup}$}}{G}\left(\overset{\lower0.5em\hbox{$\smash{\scriptscriptstyle\rightharpoonup}$}}{V}\right)={K\overset{\lower0.5em\hbox{$\smash{\scriptscriptstyle\rightharpoonup}$}}{U}}_{1}^{\left(0\right)}\left(x=0\right)+ K{\overset{\lower0.5em\hbox{$\smash{\scriptscriptstyle\rightharpoonup}$}}{U}}_{2}^{\left(0\right)}(x=0)$$

In Eq. (15), $$\overrightarrow{V}=\left({V}_{1},{V}_{2},{V}_{3}\right)$$ where the $${V}_{i}$$’s are degrees of freedom associated with the extrinsic oscillators at the ends of rods, *i* = 1,2,3. $${\omega }_{R}^{2}$$ is the characteristic frequency of an oscillator. $${\varepsilon }_{R}\overset{\lower0.5em\hbox{$\smash{\scriptscriptstyle\rightharpoonup}$}}{G}\left(\overset{\lower0.5em\hbox{$\smash{\scriptscriptstyle\rightharpoonup}$}}{V}\right)$$ represents the nonlinear behavior oscillators. The terms $${K\overset{\lower0.5em\hbox{$\smash{\scriptscriptstyle\rightharpoonup}$}}{U}}_{1}^{\left(0\right)}\left(x=0\right)+ K{\overset{\lower0.5em\hbox{$\smash{\scriptscriptstyle\rightharpoonup}$}}{U}}_{2}^{\left(0\right)}(x=0)$$ represent the driving force on the oscillators due to the linear displacement fields of the array of waveguides at their ends, *x* = 0. $$K$$ is some proportionality constant that converts displacement of the end of a waveguide into a driving force on the oscillator. Let us rewrite Eq. (15) for one oscillator “$$i$$”:

16 $$\left(\frac{{d}^{2}}{{dt}^{2}}+{\omega }_{R}^{2}+{\mu }_{R}\frac{d}{dt}\right){V}_{i}+{\varepsilon }_{R}{V}_{i}^{Q}={f}_{i}^{(1)}{e}^{i{\omega }_{1}t}+{f}_{i}^{(2)}{e}^{i{\omega }_{2}t}$$

where $${f}_{i}^{(1)}=K{C}_{i}^{(1)}{e}^{i{\varphi }_{i}^{(1)}}$$ and $${f}_{i}^{(2)}=K{C}_{i}^{(2)}{e}^{i{\varphi }_{i}^{(2)}}$$ where $${C}_{i}^{(1)}$$, $${C}_{i}^{(2)}$$ and $${\varphi }_{i}^{(1)}$$, $${\varphi }_{i}^{(2)}$$ are the *i*^th^ amplitudes and phases in Eq. (14). In Eq. (16) we have made the choice of a nonlinear function $$\overset{\lower0.5em\hbox{$\smash{\scriptscriptstyle\rightharpoonup}$}}{G}\left(\overset{\lower0.5em\hbox{$\smash{\scriptscriptstyle\rightharpoonup}$}}{V}\right)$$ that acts on individual oscillators since they are not directly coupled to each other. A general power of Q is also chosen for the functional form of the nonlinear term. We now use perturbation theory to solve Eq. (16). We expand $${V}_{i}=$$ $${V}_{i}^{0}+{\varepsilon }_{R}{V}_{i}^{1}$$. To zeroth order in perturbation $${\varepsilon }_{R}$$, the equation is linear and takes the form:

17 $$\left(\frac{{d}^{2}}{{dt}^{2}}+{\omega }_{R}^{2}+{\mu }_{R}\frac{d}{dt}\right){V}_{i}^{0}={f}_{i}^{(1)}{e}^{i{\omega }_{1}t}+{f}_{i}^{(2)}{e}^{i{\omega }_{2}t}$$

and zeroth order harmonic solutions that have the same frequencies as the driving forces take the form:

18 $${V}_{i}^{0}=\frac{{f}_{i}^{(1)}}{-{\omega }_{1}^{2}+{\omega }_{R}^{2}+i{\mu }_{R}{\omega }_{1}}{e}^{i{\omega }_{1}t}+\frac{{f}_{i}^{(2)}}{-{\omega }_{2}^{2}+{\omega }_{R}^{2}+i{\mu }_{R}{\omega }_{2}}{e}^{i{\omega }_{2}t}$$

To first order Eq. (16) becomes

19 $$\left(\frac{{d}^{2}}{{dt}^{2}}+{\omega }_{R}^{2}+{\mu }_{R}\frac{d}{dt}\right){V}_{i}^{1}+{\left({V}_{i}^{0}\right)}^{Q}=0$$

By inserting Eq. (18) into the nonlinear term ${\left({V}_{i}^{0}\right)}^{Q}$ we generate a series of terms of the form $D{\left(\frac{{f}_{i}^{(1)}}{-{\omega }_{1}^{2}+{\omega }_{R}^{2}+i{\mu }_{R}{\omega }_{1}}\right)}^{p}{\left(\frac{{f}_{i}^{(2)}}{-{\omega }_{2}^{2}+{\omega }_{R}^{2}+i{\mu }_{R}{\omega }_{2}}\right)}^{q}{e}^{i(p{\omega }_{1}+q{\omega }_{2})t}$, where *D* is a proportionality constant and $p+q=Q$. Considering only one of these terms corresponding to a pair $\left\{p.q\right\}$, Eq. (19) reduces to

20 $$\left(\frac{{d}^{2}}{{dt}^{2}}+{\omega }_{R}^{2}+{\mu }_{R}\frac{d}{dt}\right){V}_{i}^{1}+D{\left(\frac{K{C}_{i}^{(1)}}{-{\omega }_{1}^{2}+{\omega }_{R}^{2}+i{\mu }_{R}{\omega }_{1}}\right)}^{p}{\left(\frac{K{C}_{i}^{(2)}}{-{\omega }_{2}^{2}+{\omega }_{R}^{2}+i{\mu }_{R}{\omega }_{2}}\right)}^{q}{e}^{i\left(p{\varphi }_{i}^{(1)}+q{\varphi }_{i}^{(2)}\right)}{e}^{i(p{\omega }_{1}+q{\omega }_{2})t}=0$$

In writing Eq. (20) we have used the expressions for ${f}_{i}^{(1)}$ and ${f}_{i}^{(2)}$ given for Eq. (16). Seeking a particular solution of Eq. (20) leads to

21 $${V}_{i}^{1}=\frac{1}{-{\left(p{\omega }_{1}+q{\omega }_{2}\right)}^{2}+{\omega }_{R}^{2}+i{\mu }_{R}(p{\omega }_{1}+q{\omega }_{2})}{\left(\frac{K}{-{\omega }_{1}^{2}+{\omega }_{R}^{2}+i{\mu }_{R}{\omega }_{1}}\right)}^{p}{\left(\frac{K}{-{\omega }_{2}^{2}+{\omega }_{R}^{2}+i{\mu }_{R}{\omega }_{2}}\right)}^{q}{\left({C}_{i}^{(1)}\right)}^{p}{\left({C}_{i}^{(2)}\right)}^{q}{e}^{i\left(p{\varphi }_{i}^{(1)}+q{\varphi }_{i}^{(2)}\right)}{e}^{i(p{\omega }_{1}+q{\omega }_{2})t}$$

The fractions in the prefactor to the exponentials in Eq. (21) are the same for each component “$i$” of ${\overrightarrow{V}}^{1}$ and add the same general phase to each component. Therefore, we have

22 $${\overrightarrow{V}}^{1}\propto \left(\begin{array}{c}{\left({C}_{1}^{(1)}\right)}^{p}{\left({C}_{1}^{(2)}\right)}^{q}{e}^{i\left(p{\varphi }_{1}^{(1)}+q{\varphi }_{1}^{(2)}\right)}\\ {\left({C}_{2}^{(1)}\right)}^{p}{\left({C}_{2}^{(2)}\right)}^{q}{e}^{i\left(p{\varphi }_{2}^{(1)}+q{\varphi }_{2}^{(2)}\right)}\\ {\left({C}_{3}^{(1)}\right)}^{p}{\left({C}_{3}^{(2)}\right)}^{q}{e}^{i\left(p{\varphi }_{3}^{(1)}+q{\varphi }_{3}^{(2)}\right)}\end{array}\right){e}^{i(p{\omega }_{1}+q{\omega }_{2})t}$$

Note that the coefficients ${\left({C}_{i}^{(1)}\right)}^{p}{\left({C}_{i}^{(2)}\right)}^{q}$ are real. The differences in phase between oscillator 2 and 1, and, 3 and 1 are then given by: ${\varphi }_{12}^{R}=p\left({\varphi }_{2}^{\left(1\right)}-{\varphi }_{1}^{\left(1\right)}\right)+q\left({\varphi }_{2}^{(2)}-{\varphi }_{1}^{(2)}\right)$ and ${\varphi }_{13}^{R}=p\left({\varphi }_{3}^{\left(1\right)}-{\varphi }_{1}^{\left(1\right)}\right)+q\left({\varphi }_{3}^{(2)}-{\varphi }_{1}^{(2)}\right)$. Theses phases are linear combinations of the phase differences in the displacement fields at the ends of the waveguides in the coupled array. The linear combination is the same as that of the nonlinear frequency $p{\omega }_{1}+q{\omega }_{2}$. Since each oscillator is physically in contact with the ends of the rods, one expects that they will contribute to the detected signals at the ends of the waveguides. The oscillators will therefore contribute a background to the phases of a phi-bit with frequency $p{f}_{1}+q{f}_{2}$ that are linear combinations of the phase differences ${\varphi }_{12}^{(1)}$, ${\varphi }_{13}^{(1)}$, ${\varphi }_{12}^{(2)}$,and ${\varphi }_{13}^{(2)}$ associated with the linear displacement of the array of waveguides. As one varies the driving frequency ${f}_{2}=\frac{{\omega }_{2}}{2\pi }$, the variations in ${\varphi }_{12}^{(2)}$,and ${\varphi }_{13}^{(2)}$ are controlled by the variations of the complex amplitudes ${A}_{n,2}\left({k}_{n}^{^{\prime}}\right)=\frac{{F}_{n}^{(2)}}{{\omega }_{0,n}^{2}\left({k}_{n}^{^{\prime}}\right)-{\omega }_{2}^{2}+i\mu {\omega }_{2}}$ as given by Eq. (10). These variations will therefore be controlled by the characteristic frequencies of the modes of vibration of the array of waveguides: ${\omega }_{0,n}^{2}\left({k}_{n}^{^{\prime}}\right)={\beta }^{2}{k}_{n}^{{^{\prime}}2}+{\alpha }^{2}{\lambda }_{n}$. Since these characteristic frequencies are known experimentally to be spaced by on the order of several *kHz* between two consecutive discrete states for the same spatial mode or for states between different spatial modes^1^, we expect the background phase differences to exhibit variations with the same frequency scale. This model is therefore consistent with the observation of the background phases in the experimental measurements. It is therefore straightforward if one wants to eliminate the extrinsic effect of nonlinear oscillators coupled to the ends of the waveguides by subtracting the background phases from the overall signals.

## Possible origin of the π jumps

Here, we seek analytical approximations to a variation on the nonlinear Eq. (1) with two-frequency excitation using frequency detuning with multiple time scale perturbation theory^13,14^.

23 $$\left[\left(\frac{{\partial }^{2}}{{\partial t}^{2}}-{\beta }^{2}\frac{{\partial }^{2}}{{\partial x}^{2}}+2\varepsilon \mu \frac{\partial }{\partial t}\right)\overleftrightarrow{I}+{\alpha }^{2}\overleftrightarrow{M}\right]\overset{\lower0.5em\hbox{$\smash{\scriptscriptstyle\rightharpoonup}$}}{U}+\varepsilon \delta \overset{\lower0.5em\hbox{$\smash{\scriptscriptstyle\rightharpoonup}$}}{G}(\overset{\lower0.5em\hbox{$\smash{\scriptscriptstyle\rightharpoonup}$}}{U})={\overset{\lower0.5em\hbox{$\smash{\scriptscriptstyle\rightharpoonup}$}}{F}}_{1}{\delta }_{x=0}\mathrm{cos}{\omega }_{1}t+{\overset{\lower0.5em\hbox{$\smash{\scriptscriptstyle\rightharpoonup}$}}{F}}_{2}{\delta }_{x=0}\mathrm{cos}{\omega }_{2}t$$

Note the additional parameter $$\delta$$ and the dependency of damping on $$\varepsilon$$. $$\overset{\lower0.5em\hbox{$\smash{\scriptscriptstyle\rightharpoonup}$}}{G}(\overset{\lower0.5em\hbox{$\smash{\scriptscriptstyle\rightharpoonup}$}}{U})$$ models nonlinearities that act on the waveguides along their length. It may therefore represent qualitatively the effect of the epoxy nonlinearity.

We consider two time scales, $${\tau }_{0}=t$$ and $${\tau }_{1}=\varepsilon t$$. We also expend the displacement field as a sum of a zeroth order (linear) and first order (nonlinear) terms as follows: $$\overset{\lower0.5em\hbox{$\smash{\scriptscriptstyle\rightharpoonup}$}}{U}={\overset{\lower0.5em\hbox{$\smash{\scriptscriptstyle\rightharpoonup}$}}{U}}^{\left(0\right)}({\tau }_{0},{\tau }_{1})+ \epsilon {\overset{\lower0.5em\hbox{$\smash{\scriptscriptstyle\rightharpoonup}$}}{U}}^{(1)}({\tau }_{0},{\tau }_{1})$$. The first order and second order time derivatives take the form:

24a $$\frac{\partial \overset{\lower0.5em\hbox{$\smash{\scriptscriptstyle\rightharpoonup}$}}{U}}{\partial t}=\frac{\partial {\overset{\lower0.5em\hbox{$\smash{\scriptscriptstyle\rightharpoonup}$}}{U}}^{(0)}}{\partial {\tau }_{0}}+\varepsilon \left(\frac{\partial {\overset{\lower0.5em\hbox{$\smash{\scriptscriptstyle\rightharpoonup}$}}{U}}^{(1)}}{\partial {\tau }_{0}}+\frac{\partial {\overset{\lower0.5em\hbox{$\smash{\scriptscriptstyle\rightharpoonup}$}}{U}}^{(0)}}{\partial {\tau }_{1}}\right)$$

24b $$\frac{{\partial }^{2}\overset{\lower0.5em\hbox{$\smash{\scriptscriptstyle\rightharpoonup}$}}{U}}{{\partial t}^{2}}=\frac{{\partial }^{2}{\overset{\lower0.5em\hbox{$\smash{\scriptscriptstyle\rightharpoonup}$}}{U}}^{(0)}}{{\partial {\tau }_{0}}^{2}}+\varepsilon \left(\frac{{\partial }^{2}{\overset{\lower0.5em\hbox{$\smash{\scriptscriptstyle\rightharpoonup}$}}{U}}^{(1)}}{{\partial {\tau }_{0}}^{2}}+2\frac{{\partial }^{2}{\overset{\lower0.5em\hbox{$\smash{\scriptscriptstyle\rightharpoonup}$}}{U}}^{(0)}}{\partial {\tau }_{1}\partial {\tau }_{0}}\right)$$

The wave equation to 0th order in $\varepsilon$ is effectively the linear equation:

25 $$\left[\left(\frac{{\partial }^{2}}{{\partial {\tau }_{0}}^{2}}-{\beta }^{2}\frac{{\partial }^{2}}{{\partial x}^{2}}\right)\overleftrightarrow{I}+{\alpha }^{2}\overleftrightarrow{M}\right]{\overset{\lower0.5em\hbox{$\smash{\scriptscriptstyle\rightharpoonup}$}}{U}}^{(0)}={\overset{\lower0.5em\hbox{$\smash{\scriptscriptstyle\rightharpoonup}$}}{F}}_{1}{\delta }_{x=0}\mathrm{cos}{\omega }_{1}{\tau }_{0}+{\overset{\lower0.5em\hbox{$\smash{\scriptscriptstyle\rightharpoonup}$}}{F}}_{2}{\delta }_{x=0}\mathrm{cos}{\omega }_{2}{\tau }_{0}$$

Note that there is no damping coefficient in Eq. (25) as the effect of damping is now included into the first order equation. We will see later that the phase associated with damping included now in the 1st order equation will come back as a correction to the complex amplitude of the 0th order solution.

To 1st order, the wave equation becomes:

26 $$\left[\left(\frac{{\partial }^{2}}{{\partial {\tau }_{o}}^{2}}-{\beta }^{2}\frac{{\partial }^{2}}{{\partial x}^{2}}\right)\overleftrightarrow{I}+{\alpha }^{2}\overleftrightarrow{M}\right]{\overset{\lower0.5em\hbox{$\smash{\scriptscriptstyle\rightharpoonup}$}}{U}}^{(1)}=-2\frac{{\partial }^{2}{\overset{\lower0.5em\hbox{$\smash{\scriptscriptstyle\rightharpoonup}$}}{U}}^{\left(0\right)}}{\partial {\tau }_{1}\partial {\tau }_{0}}-2\mu \frac{\partial {\overset{\lower0.5em\hbox{$\smash{\scriptscriptstyle\rightharpoonup}$}}{U}}^{\left(0\right)}}{\partial {\tau }_{0}}-\delta \overset{\lower0.5em\hbox{$\smash{\scriptscriptstyle\rightharpoonup}$}}{G}({\overset{\lower0.5em\hbox{$\smash{\scriptscriptstyle\rightharpoonup}$}}{U}}^{\left(0\right)})$$

Solutions to Eq. (25) include the sum of a solution of the homogeneous equation (i.e., without external driving) and a particular solution resulting from the driving. We have already obtained the particular solution of the 0^th^ order equation (linear equation) in Section “Experimental results”. The complete solution takes therefore the form:

27a $${\overset{\lower0.5em\hbox{$\smash{\scriptscriptstyle\rightharpoonup}$}}{U}}^{(0)}=\sum_{n=1}^{3}{\overset{\lower0.5em\hbox{$\smash{\scriptscriptstyle\rightharpoonup}$}}{E}}_{n}{u}_{0,n}$$

with

27b $${u}_{0,n}=\left(\sum_{{k}_{n}}{\Lambda }_{n}({k}_{n},{\tau }_{1}){e}^{i{k}_{n}x}{e}^{i{\omega }_{0,n}{\tau }_{0}}+\sum_{{k}_{n}}{\Lambda }_{n}^{*}({k}_{n},{\tau }_{1}){e}^{-i{k}_{n}x}{e}^{-i{\omega }_{0,n}{\tau }_{0}}\right)+\left(\sum_{{k}_{n}}{A}_{n,1}({k}_{n}){e}^{i{k}_{n}x}{e}^{i{\omega }_{1}{\tau }_{0}}+\sum_{{k}_{n}}{A}_{n,2}({k}_{n}){e}^{i{k}_{n}x}{e}^{i{\omega }_{2}{\tau }_{0}}+\sum_{{k}_{n}}{A}_{n,1}({k}_{n}){e}^{-i{k}_{n}x}{e}^{-i{\omega }_{1}{\tau }_{0}}+\sum_{{k}_{n}}{A}_{n,2}({k}_{n}){e}^{-i{k}_{n}x}{e}^{-i{\omega }_{2}{\tau }_{0}}\right)$$

Here, the quantities ${A}_{n,l}\left({k}_{n}\right)=\frac{{F}_{n}^{(l)}}{{\omega }_{0,n}^{2}\left({k}_{n}\right)-{\omega }_{l}^{2}}$ are now real. The star in Eq. (27b) stands for the complex conjugate. For the sake of simplifying the notation, we have dropped here the different labelling of the same wave numbers ${k}_{n}$ and ${k}_{n}^{^{\prime}}$ used in Section “Experimental results”. The first two bracket in Eq. (27a) correspond to the solution to the homogeneous equation with ${\omega }_{0,n}$ defined in Section “Experimental results”. The second bracket correspond to the particular solution. Both brackets now include the complex solutions and their complex conjugate to account for the real nature of the displacement and the real form of the driving forces $\mathrm{cos}{\omega }_{1}{\tau }_{0}$ and $\mathrm{cos}{\omega }_{2}{\tau }_{0}$.

We now chose a form for the term $$\overset{\lower0.5em\hbox{$\smash{\scriptscriptstyle\rightharpoonup}$}}{G}({\overset{\lower0.5em\hbox{$\smash{\scriptscriptstyle\rightharpoonup}$}}{U}}^{\left(0\right)})$$ that enables to proceed analytically:

28 $$\overset{\lower0.5em\hbox{$\smash{\scriptscriptstyle\rightharpoonup}$}}{G}({\overset{\lower0.5em\hbox{$\smash{\scriptscriptstyle\rightharpoonup}$}}{U}}^{\left(0\right)})=\sum_{n=1}^{3}{\overset{\lower0.5em\hbox{$\smash{\scriptscriptstyle\rightharpoonup}$}}{E}}_{n}{\left({u}_{0,n}\right)}^{3}$$

This form assumes that the spatial modes, $${\overset{\lower0.5em\hbox{$\smash{\scriptscriptstyle\rightharpoonup}$}}{E}}_{n}$$, do not interact with each other. However, for each spatial mode, the plane wave modes, $${e}^{i{k}_{n}x}$$, interact with each other. We use a third order nonlinearity for the sake of analytical tractability. The behavior of the system for even orders of nonlinearity and higher orders of nonlinearity, *Q,* will be discussed at the end of this section.

Defining $${\overset{\lower0.5em\hbox{$\smash{\scriptscriptstyle\rightharpoonup}$}}{U}}^{(1)}=\sum_{n=1}^{3}{\overset{\lower0.5em\hbox{$\smash{\scriptscriptstyle\rightharpoonup}$}}{E}}_{n}{u}_{1,n}$$ and using Eq. (28), we can rewrite Eq. (26) as a set of three equation, each one corresponding to a different spatial mode:

29 $$\left[\frac{{\partial }^{2}}{{\partial {\tau }_{o}}^{2}}-{\beta }^{2}\frac{{\partial }^{2}}{{\partial x}^{2}}+{\alpha }^{2}{\lambda }_{n}\right]{u}_{1,n}=-2\frac{{\partial }^{2}{u}_{0,n}}{\partial {\tau }_{1}\partial {\tau }_{0}}-2\mu \frac{\partial {u}_{0,n}}{\partial {\tau }_{0}}-\delta {\left({u}_{0,n}\right)}^{3}$$

To calculate ${\left({u}_{0,n}\right)}^{3}$, we rewrite Eq. (27b) as

30 $${u}_{0,n}=\sum_{{k}_{n}}{\upeta }_{n}{e}^{i{k}_{n}x}+\sum_{{k}_{n}}{\eta }_{n}^{*}{e}^{-i{k}_{n}x}$$

with ${\upeta }_{n}={\Lambda }_{n}{e}^{i{\omega }_{0,n}{\tau }_{0}}+{A}_{n,1}{e}^{i{\omega }_{1}{\tau }_{0}}+{A}_{n,2}{e}^{i{\omega }_{2}{\tau }_{0}}$. We this, we rewrite the cubic term as

$${\left({u}_{0,n}\right)}^{3}=\left(\sum_{{k}_{n}}{\upeta }_{n}{e}^{i{k}_{n}x}+\sum_{{k}_{n}}{\eta }_{n}^{*}{e}^{-i{k}_{n}x}\right)\left(\sum_{{k}_{n}^{^{\prime}}}{\eta }_{n}^{^{\prime}}{e}^{i{k}_{n}^{^{\prime}}x}+\sum_{{k}_{n}^{^{\prime}}}{\eta }_{n}^{{^{\prime}}*}{e}^{-i{k}_{n}^{^{\prime}}x}\right)\left(\sum_{{k}_{n}^{{^{\prime}}{^{\prime}}}}{\eta }_{n}^{{^{\prime}}{^{\prime}}}{e}^{i{k}_{n}^{{^{\prime}}{^{\prime}}}x}+\sum_{{k}_{n}}{\eta }_{n}^{{^{\prime}}{^{\prime}}*}{e}^{-i{k}_{n}^{{^{\prime}}{^{\prime}}}x}\right)$$

In this expression, since we have the produce of three summations over the wave number, we reintroduced three different representations of the same wavenumbers, ${k}_{n}$, ${k}_{n}^{^{\prime}}$, ${k}_{n}^{{^{\prime}}{^{\prime}}}$. After several algebraic manipulation and the recognition that several multiple sums are identically the same since ${k}_{n}$, ${k}_{n}^{^{\prime}}$, ${k}_{n}^{{^{\prime}}{^{\prime}}}$ represent the same wave numbers, we obtain:

31 $${\left({u}_{0,n}\right)}^{3}=\sum_{{k}_{n}}\sum_{{k}_{n}^{^{\prime}}}\sum_{{k}_{n}^{{^{\prime}}{^{\prime}}}}\left\{{\upeta }_{n}{\eta }_{n}^{^{\prime}}{\eta }_{n}^{{^{\prime}}{^{\prime}}}{e}^{i\left({k}_{n}+ {k}_{n}^{^{\prime}}+{k}_{n}^{{^{\prime}}{^{\prime}}}\right)x}+3{\upeta }_{n}{\eta }_{n}^{{^{\prime}}*}{\eta }_{n}^{{^{\prime}}{^{\prime}}}{e}^{i\left({k}_{n}- {k}_{n}^{^{\prime}}+{k}_{n}^{{^{\prime}}{^{\prime}}}\right)x}+3{\upeta }_{n}{\eta }_{n}^{{^{\prime}}*}{\eta }_{n}^{{^{\prime}}{^{\prime}}*}{e}^{i\left({k}_{n}- {k}_{n}^{^{\prime}}-{k}_{n}^{{^{\prime}}{^{\prime}}}\right)x}+{\eta }_{n}^{*}{\eta }_{n}^{{^{\prime}}*}{\eta }_{n}^{{^{\prime}}{^{\prime}}*}{e}^{i\left(-{k}_{n}- {k}_{n}^{^{\prime}}-{k}_{n}^{{^{\prime}}{^{\prime}}}\right)x}\right\}$$

We seek first order displacement solutions

32 $${u}_{1,n}=\left(\sum_{{k}_{n}}{\mathrm{\rm B}}_{n}({k}_{n},{\tau }_{0}){e}^{i{k}_{n}x}{e}^{i{\omega }_{0,n}{\tau }_{0}}+\sum_{{k}_{n}}{\mathrm{\rm B}}_{n}^{*}({k}_{n},{\tau }_{0}){e}^{-i{k}_{n}x}{e}^{-i{\omega }_{0,n}{\tau }_{0}}\right)$$

Inserting Eqs. (31) and (32) into Eq. (29), multiplying the left and tight sides of the equal sign by ${e}^{i{k}_{n}^{s}x}$ and integrating over *x*, selects a specific ${k}_{n}^{s}$ yielding the equation:

33 $$\left[\frac{{\partial }^{2}{\mathrm{\rm B}}_{n}}{{\partial {\tau }_{o}}^{2}}+{\beta }^{2}{\left({k}_{n}^{s}\right)}^{2}{\mathrm{\rm B}}_{n}+{\alpha }^{2}{\lambda }_{n}{\mathrm{\rm B}}_{n}\right]+\left[c.c.\right]=-2\frac{{\partial }^{2}{\upeta }_{n}({k}_{n}^{s})}{\partial {\tau }_{1}\partial {\tau }_{0}}-2\frac{{\partial }^{2}{\eta }_{n}^{*}({-k}_{n}^{s})}{\partial {\tau }_{1}\partial {\tau }_{0}}-2\mu \frac{\partial {\upeta }_{n}({k}_{n}^{s})}{\partial {\tau }_{0}}-2\mu \frac{\partial {\eta }_{n}^{*}(-{k}_{n}^{s})}{\partial {\tau }_{0}}-\delta \sum_{{k}_{n}}\sum_{{k}_{n}^{^{\prime}}}\sum_{{k}_{n}^{{^{\prime}}{^{\prime}}}}\left\{{\upeta }_{n}{\eta }_{n}^{^{\prime}}{\eta }_{n}^{{^{\prime}}{^{\prime}}}{\delta }_{{k}_{n,}^{s}{k}_{n}+ {k}_{n}^{^{\prime}}+{k}_{n}^{{^{\prime}}{^{\prime}}}}+3{\upeta }_{n}{\eta }_{n}^{{^{\prime}}*}{\eta }_{n}^{{^{\prime}}{^{\prime}}}{\delta }_{{k}_{n}^{s}{,k}_{n}- {k}_{n}^{^{\prime}}+{k}_{n}^{{^{\prime}}{^{\prime}}}}+3{\upeta }_{n}{\eta }_{n}^{{^{\prime}}*}{\eta }_{n}^{{^{\prime}}{^{\prime}}*}{\delta }_{{k}_{n}^{s},{k}_{n}\pm {k}_{n}^{^{\prime}}-{k}_{n}^{{^{\prime}}{^{\prime}}}}+{\eta }_{n}^{*}{\eta }_{n}^{{^{\prime}}*}{\eta }_{n}^{{^{\prime}}{^{\prime}}*}{\delta }_{{k}_{n}^{s}{,-k}_{n}- {k}_{n}^{^{\prime}}-{k}_{n}^{{^{\prime}}{^{\prime}}}}\right\}$$

For this we have used the fact that $\int dx{e}^{i\left({k}^{s}-k\right)x}={\delta }_{{k}^{s},k}$, the Kronecker symbol. In Eq. (33) “[c.c.]” stands for the complex conjugate of the square bracket.

Using the definition of ${\upeta }_{n}$ and its complex conjugate, we calculate

$$-2\mu \frac{\partial {\upeta }_{n}({k}_{n}^{s})}{\partial {\tau }_{0}}=-2\mu \left(i{\omega }_{0,n}^{s}{\Lambda }_{n}{e}^{i{\omega }_{0,n}^{s}{\tau }_{0}}+{A}_{n,1}i{\omega }_{1}{e}^{i{\omega }_{1}{\tau }_{0}}+{A}_{n,2}{i{\omega }_{2}e}^{i{\omega }_{2}{\tau }_{0}}\right)$$

$$-2\mu \frac{\partial {\eta }_{n}^{*}({-k}_{n}^{s})}{\partial {\tau }_{0}}=-2\mu \left(-i{\omega }_{0,n}^{s}{\Lambda }_{n}^{*}{e}^{-i{\omega }_{0,n}^{s}{\tau }_{0}}-{A}_{n,1}{i{\omega }_{1}e}^{-i{\omega }_{1}{\tau }_{0}}-{A}_{n,2}{i{\omega }_{2}e}^{-i{\omega }_{2}{\tau }_{0}}\right)$$

$$-2\frac{{\partial }^{2}{\upeta }_{n}\left({k}_{n}^{s}\right)}{\partial {\tau }_{1}\partial {\tau }_{0}}=-2i{\omega }_{0,n}^{s}\frac{\partial {\Lambda }_{n}}{\partial {\tau }_{1}}{e}^{i{\omega }_{0,n}^{s}{\tau }_{0}}$$

$$-2\frac{{\partial }^{2}{\eta }_{n}^{*}\left({-k}_{n}^{s}\right)}{\partial {\tau }_{1}\partial {\tau }_{0}}=+2i{\omega }_{0,n}^{s}\frac{\partial {\Lambda }_{n}^{*}}{\partial {\tau }_{1}}{e}^{-i{\omega }_{0,n}^{s}{\tau }_{0}}$$

These terms constitute secular terms that need to be eliminated through corresponding terms in the triple summation of Eq. (33). Seeking all the possible combinations of positive and negative ${k}_{n}$, ${k}_{n}^{^{\prime}}$, ${k}_{n}^{{^{\prime}}{^{\prime}}}$, that would lead to secular terms in ${e}^{i{\omega }_{0,n}^{s}{\tau }_{0}}$, we obtain the terms:

34 $${{T}_{1}=\left(3{\Lambda }_{n}({k}_{n}^{s})\sum_{{k}_{n}}{\Lambda }_{n}\left({k}_{n}\right){\Lambda }_{n}^{*}\left({k}_{n}\right)+3\times 2{\Lambda }_{n}({k}_{n}^{s})\sum_{{k}_{n}}\left({A}_{n,1}^{2}+{A}_{n,2}^{2}\right)\right)e}^{i{\omega }_{0,n}^{s}{\tau }_{0}}$$

We now seek the terms without ${e}^{i{\omega }_{0,n}^{s}{\tau }_{0}}$, considering for instance the terms multiplied by the exponential, ${e}^{i(2{\omega }_{1}+{\omega }_{2}){\tau }_{0}}$, we get the following:

35 $${T}_{2}=\sum_{{k}_{n}}\left(2{A}_{n,1}({k}_{n}^{s}){A}_{n,1}({k}_{n}){A}_{n,2}\left({k}_{n}\right)+{A}_{n,2}({k}_{n}^{s}){A}_{n,1}({k}_{n}){A}_{n,1}\left({k}_{n}\right)\right){e}^{i(2{\omega }_{1}+{\omega }_{2}){\tau }_{0}}$$

We introduce a detuning parameter: $\sigma =\frac{1}{\varepsilon }\left(2{\omega }_{1}+{\omega }_{2}-{\omega }_{0,n}^{s}\right)$, Eq. (35) is now rewritten as

36 $${T}_{2}=\sum_{{k}_{n}}\left(2{A}_{n,1}({k}_{n}^{s}){A}_{n,1}({k}_{n}){A}_{n,2}\left({k}_{n}\right)+{A}_{n,2}({k}_{n}^{s}){A}_{n,1}({k}_{n}){A}_{n,1}\left({k}_{n}\right)\right){e}^{i\sigma {\tau }_{1}}{e}^{i{\omega }_{0,n}^{s}{\tau }_{0}}$$

Adding all the secular terms in ${e}^{i{\omega }_{0,n}^{s}{\tau }_{0}}$ present in the right-hand side of Eq. (33) leads to the secular equation:

37 $$2\mu i{\omega }_{0,n}^{s}{\Lambda }_{n}\left({k}_{n}^{s}\right)+2i{\omega }_{0,n}^{s}{\Lambda }_{n}^{^{\prime}}\left({k}_{n}^{s}\right)+\delta \left(3{\Lambda }_{n}({k}_{n}^{s})\sum_{{k}_{n}}{\Lambda }_{n}\left({k}_{n}\right){\Lambda }_{n}^{*}\left({k}_{n}\right)+3\times 2{\Lambda }_{n}({k}_{n}^{s})\sum_{{k}_{n}}\left({A}_{n,1}^{2}+{A}_{n,2}^{2}\right)\right)+\delta \sum_{{k}_{n}}\left(2{A}_{n,1}({k}_{n}^{s}){A}_{n,1}({k}_{n}){A}_{n,2}\left({k}_{n}\right)+{A}_{n,2}({k}_{n}^{s}){A}_{n,1}({k}_{n}){A}_{n,1}\left({k}_{n}\right)\right){e}^{i\sigma {\tau }_{1}}=0$$

In Eq. (37) ${\Lambda }_{n}^{^{\prime}}=\frac{\partial {\Lambda }_{n}}{\partial {\tau }_{1}}$. In the subsequent equations a prime indicates a time derivative with respect to ${\tau }_{1}$

We now define ${\Lambda }_{n}\left(k\right)=\frac{1}{2}a(k){e}^{ib(k,{\tau }_{1})}$, such that the secular equation reduces to

38 $$\mu {i\omega }_{0,n}^{s}a\left({k}_{n}^{s}\right)+i{\omega }_{0,n}^{s}i{b}^{^{\prime}}\left({k}_{n}^{s},{\tau }_{1}\right)a\left({k}_{n}^{s}\right)+\frac{3}{2}\delta a\left({k}_{n}^{s}\right)\sum_{{k}_{n}}\frac{1}{4}{a}^{2}\left({k}_{n}^{s}\right)+\delta a\left({k}_{n}^{s}\right){\Gamma }_{1} {\omega }_{0,n}^{s}+\delta {\Gamma }_{2} {\omega }_{0,n}^{s}{e}^{i\sigma {\tau }_{1}}{e}^{-ib(k,{\tau }_{1})}=0$$

where ${\Gamma }_{1}=\frac{3}{{\omega }_{0,n}^{s}}\sum_{{k}_{n}}\left({A}_{n,1}^{2}+{A}_{n,2}^{2}\right)$ and ${\Gamma }_{2}=\frac{1}{{\omega }_{0,n}^{s}}\sum_{{k}_{n}}\left(2{A}_{n,1}({k}_{n}^{s}){A}_{n,1}({k}_{n}){A}_{n,2}\left({k}_{n}\right)+{A}_{n,2}({k}_{n}^{s}){A}_{n,1}({k}_{n}){A}_{n,1}\left({k}_{n}\right)\right)$. Both ${\Gamma }_{1}$ and ${\Gamma }_{2}$ are real quantities. We further define $\varsigma =\sigma {\tau }_{1}-b$ such that ${b}^{{^{\prime}}\left({k}_{n}^{s},{\tau }_{1}\right)}=\sigma -\varsigma {^{\prime}}$.

We now regroup the real and imaginary terms of Eq. (38) into two separate equation and seek only steady state solution by setting all primed quantities (time derivatives with respect to ${\tau }_{1})$ to zero. We finally get the set of equations:

39a $$a\left({k}_{n}^{s}\right)+\frac{\delta {\Gamma }_{2}}{\mu }\mathrm{sin}\varsigma =0$$

39b $$\left[\sigma -\delta {\Gamma }_{1}-\frac{3\delta }{{\omega }_{0,n}^{s}}\sum_{{k}_{n}}\frac{1}{8}{a}^{2}\left({k}_{n}\right)\right]-\delta {\Gamma }_{2}\mathrm{cos}\varsigma =0$$

Equation (39a) and (39b) couples the amplitude of the mode of interest ($${k}_{n}^{s})$$ with the amplitude of all other modes ($${k}_{n}$$). This equation can be simplified if we consider the self-interaction of mode $${k}_{n}^{s}$$. In that case the last term in the square bracket reduces to $$\frac{3\delta {a}^{2}\left({k}_{n}^{s}\right)}{{8\omega }_{0,n}^{s}}$$. Note also that in considering the self interaction, one need to reduce the summation over $${k}_{n}$$ in the definition of $${\Gamma }_{2}$$ to the terms in $${k}_{n}^{s}$$.

We can get the amplitude frequency response due to self-interaction by eliminating the phase $\varsigma$ using the trigonometric relation: ${\mathrm{sin}}^{2}\varsigma +{\mathrm{cos}}^{2}\varsigma =1.$ That response takes the form:

40 $$\sigma =\delta {\Gamma }_{1}+\frac{3\delta {a}^{2}\left({k}_{n}^{s}\right)}{{8\omega }_{0,n}^{s}}\pm {\left(\frac{{\left(\delta {\Gamma }_{2}\right)}^{2}}{{a}^{2}\left({k}_{n}^{s}\right)}-{\mu }^{2}\right)}^{1/2}$$

**Figure 7:**
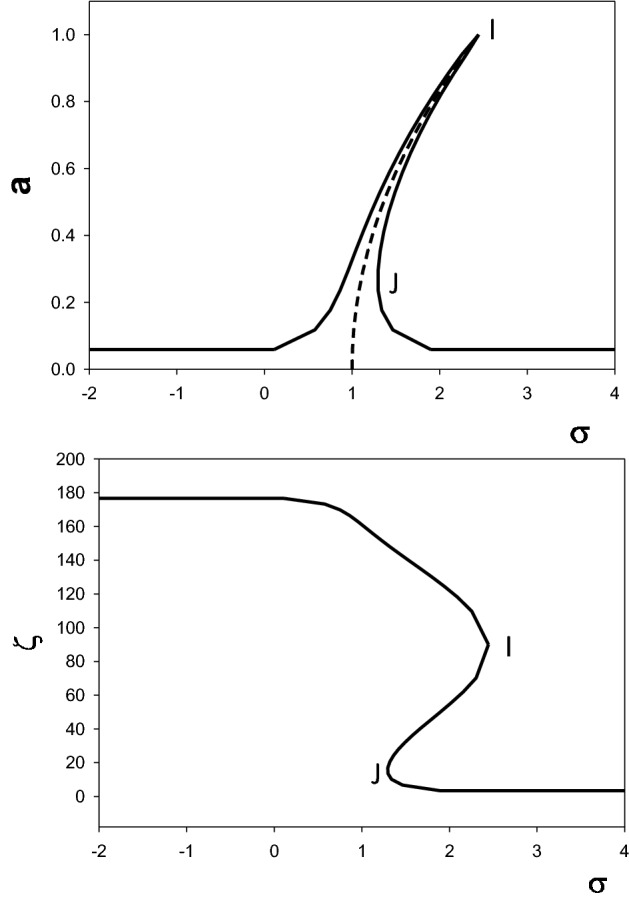
Schematic illustration of the amplitude frequency response (top) and phase frequency response (bottom) of the forced array of coupled waveguides due to self-interaction. The frequency $\sigma =\frac{1}{\varepsilon }\left(2{\omega }_{1}+{\omega }_{2}-{\omega }_{0,n}^{s}\right)$ is normalized to $\delta {\Gamma }_{1}$. The amplitude is normalized to ${a}_{m}$. The dashed line is the backbone curve for the amplitude. The phase is in degrees. The points I and J are the limit of stability when increasing or decreasing frequency, respectively.

This result is reminiscent of the amplitude frequency response of the forced Duffing oscillator^15^. Here, we consider the features of the Duffing oscillators which are relevant to explaining the experimental behavior observed in Section “Experimental results”. $a\left({k}_{n}^{s}\right)$ solution of Eq. (40) is the correction to the amplitude ${\Lambda }_{n}\left({k}_{n}^{s}\right)$ solution to the homogenous 0th order wave equation as a result of the nonlinear perturbation. In some frequency range, $\sigma$, the amplitude frequency response may not be a single-valued function. The amplitude may show overhangs to the high-frequency or low frequency depending on the sign of delta. It is centered on the backbone curve given by $\sigma =\delta {\Gamma }_{1}+\frac{3\delta {a}^{2}\left({k}_{n}^{s}\right)}{{8\omega }_{0,n}^{s}}$ where the amplitude decays to zero when $\sigma =\delta {\Gamma }_{1}$. The maximum value of the amplitude is ${a}_{m}=\frac{\left|\delta {\Gamma }_{2}\right|}{\mu }$. We obtain the phase from Eq. (39a) as $\mathrm{sin}\varsigma =sgn\left(\delta {\Gamma }_{2}\right)\frac{a}{{a}_{m}}$. In Fig. 7 we illustrate schematically the behavior of the amplitude and phase as functions of the frequency $\sigma =\frac{1}{\varepsilon }\left(2{\omega }_{1}+{\omega }_{2}-{\omega }_{0,n}^{s}\right)$.

The upper overhanging side of the response leads to a jump at ${a}_{m}$ when the frequency is slowly increased. The lower overhanging side of the response is unstable, one anticipates that when the driving frequency is slowly decreased, a jump is occurring when the point J on the Fig. 7 is reached. The instability leads to a hysteresis behavior. When increasing frequency, the phase is expected to jump by π/2. Upon decreasing frequency, the phase jump will approach π. However, since the magnitude of the phase jumps will depend on the parameters $\delta$ and $\mu$. The parameter $\delta$ which determines the strength of the nonlinearity controls the amount of overhang in the frequency domain of the amplitude. The parameter $\mu$ characterizing damping controls the width of the amplitude frequency response peak. We therefore anticipate small values of $\delta$ and $\mu$ in the case of epoxy as a source of damping and nonlinearity, leading to small overhangs and narrow amplitude and phase frequency responses. In that case, one should observe phase jumps on the order of π with very narrow hysteresis in frequency. There may be cases of nonlinearity leading to imperceptible hysteresis.

So far, we have illustrated the effect of cubic nonlinearity on the amplitude $${u}_{0,n}$$ in forming a super-harmonic nonlinear resonance when the quantity $$2{\omega }_{1}+{\omega }_{2}-{\omega }_{0,n}^{s}$$ approaches $$\delta {\Gamma }_{1}$$. Similar behavior may occur for other odd or even orders of nonlinearity. The mathematics, however, becomes intractable even for analytical approximate solutions. Considering an order of nonlinearity $$Q=p+q$$, in the event a nonlinear resonance occurs in the vicinity of the frequency $${\omega }_{0,n}^{s}\sim p{\omega }_{1}+{q\omega }_{2}$$, one expects phase jumps in $${\Lambda }_{n}$$ and therefore, $${u}_{0,n}$$ [see Eq. (27b)]. These jumps will also transfer to $${\overset{\lower0.5em\hbox{$\smash{\scriptscriptstyle\rightharpoonup}$}}{U}}^{(0)}$$ through Eq. (27a). Measurements of the phase differences $${\varphi }_{12}$$ and $${\varphi }_{13}$$ will display these nonlinear phase jumps. All the observations made from the approximate analytical solution above are consistent with the experimental observations of Figs. 3,4, and 5.

## Predictable quantum-like operations using phase jumps

Let us now consider the application of the observed nonlinear behavior to information processing. We recall that a logical phi-bit “*j*” is characterized by a displacement field measured at the ends of the rods taking the general form: $${\overrightarrow{U}}_{(j)}=\left(\begin{array}{c}{\widehat{c}}_{2}{e}^{i{\varphi }_{12}^{(j)}}\\ {\widehat{c}}_{3}{e}^{i{\varphi }_{13}^{(j)}}\end{array}\right){e}^{i{\omega }^{(j)}t}$$. Let us reconsider phi-bits *j* = *A* and *j* = *B* of Section “Experimental results”. The nonlinear frequencies of phi-bits A and B are $5{f}_{1}-2{f}_{2}$ and $4{f}_{1}-2{f}_{2}$, respectively with ${f}_{1}=62 \, {\text{kHz}}$ and ${f}_{2}=70\, {\text{kHz}}-\Delta \upsilon$ with $\Delta \upsilon$ varying in increments of 50 Hz until it reaches 62 kHz. $\Delta \upsilon$ serves therefore as a parameter to tune the state of the phi-bits. We redefine the state of the two logical phi-bits in terms of phase only by constructing the normalized state vectors:

41 $${\psi }^{(A)}=\frac{1}{\sqrt{2}}\left(\begin{array}{c}{e}^{i{\varphi }_{12}^{(A)}}\\ {e}^{i{\varphi }_{13}^{(A)}}\end{array}\right) and {\psi }^{(B)}=\frac{1}{\sqrt{2}}\left(\begin{array}{c}{e}^{i{\varphi }_{12}^{(B)}}\\ {e}^{i{\varphi }_{13}^{(B)}}\end{array}\right)$$

These state vectors live in single phi-bit 2D Hilbert space, with basis $$\left\{\left(\begin{array}{c}1\\ 0\end{array}\right),\left(\begin{array}{c}0\\ 1\end{array}\right)\right\}$$. The quantities ${e}^{i{\varphi }_{12}^{(j)}}$ and ${e}^{i{\varphi }_{13}^{(j)}}$, $j=A,B$, represent the complex coefficient in a linear combination of the 2D basis vectors. We now construct a state vector for a composite system composed of the two phi-bit. This state vector is represented as the tensor product:

42 $${\Psi }^{(AB)}={\psi }^{(A)}\otimes {\psi }^{(B)}=\frac{1}{2}\left(\begin{array}{c}{e}^{i{\varphi }_{12}^{(A)}}{e}^{i{\varphi }_{12}^{(B)}}\\ {e}^{i{\varphi }_{12}^{(A)}}{e}^{i{\varphi }_{13}^{(B)}}\\ {e}^{i{\varphi }_{13}^{(A)}}{e}^{i{\varphi }_{12}^{(B)}}\\ {e}^{i{\varphi }_{13}^{(A)}}{e}^{i{\varphi }_{13}^{(B)}}\end{array}\right)$$

This state vector lives in the 4D Hilbert space, tensor product of two 2D single phi-bit Hilbert spaces. The basis of that 4D space is composed of the tensor products of the basis vectors of the 2D spaces. We can then define new representations of the *AB* phi-bit system by applying a unitary transformation. Let us consider the following block diagonal unitary matrix:

43 $$\overleftrightarrow{T}=\frac{1}{\sqrt{2}}\left(\begin{array}{cccc}{e}^{-i{\varphi }_{12}^{(A)}}& {e}^{-i{\varphi }_{13}^{(B)}}& 0& 0\\ {-e}^{-i{\varphi }_{12}^{(A)}}& {e}^{-i{\varphi }_{13}^{(B)}}& 0& 0\\ 0& 0& {e}^{-i{\varphi }_{13}^{(A)}}& {e}^{-i{\varphi }_{12}^{(B)}}\\ 0& 0& {e}^{-i{\varphi }_{13}^{(A)}}& {-e}^{-i{\varphi }_{12}^{(B)}}\end{array}\right)$$

Application of this transformation to ${\Psi }^{(AB)}$, leads to the normalized state vector

44 $$\overleftrightarrow{T}{\Psi }^{(AB)}={\Phi }^{(AB)}=\frac{1}{2\sqrt{2}}\left(\begin{array}{c}{e}^{i{\varphi }_{12}^{(A)}}+{e}^{i{\varphi }_{12}^{(B)}}\\ {e}^{i{\varphi }_{12}^{(A)}}-{e}^{i{\varphi }_{12}^{(B)}}\\ {e}^{i{\varphi }_{13}^{(A)}}+{e}^{i{\varphi }_{13}^{(B)}}\\ {e}^{i{\varphi }_{13}^{(A)}}-{e}^{i{\varphi }_{13}^{(B)}}\end{array}\right)$$

**Figure 8:**
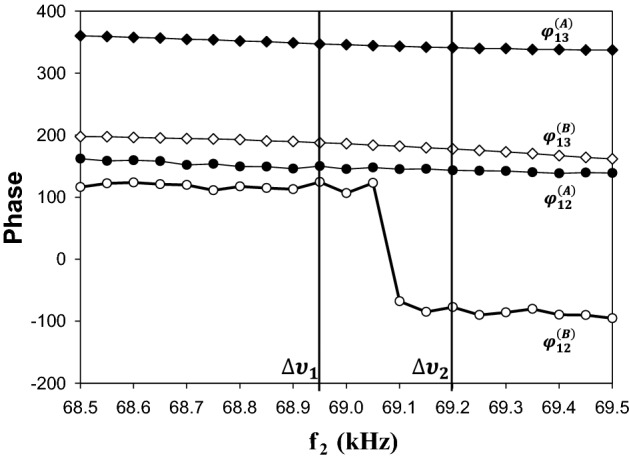
Phases ${\varphi }_{12}$ (open symbols) and ${\varphi }_{13}$ (closed symbols) measured for the phi-bits *A* and *B* as functions of ${f}_{2}=70\, {\text{kHz}}-\Delta \upsilon$ where $\Delta \upsilon$ is a frequency tuning parameter. A change of tuning parameter from ${\Delta \upsilon }_{1}$ to ${\Delta \upsilon }_{2}$ corresponds to the action of a two phi-bit CNOT-like gate. See text for details. The frequencies are in kHz and the phase in degrees.

$\overleftrightarrow{T}$ is one out of an infinite number of unitary transformations that produce different representations of the state of the *AB* composite system. Let us now see how ${\Phi }^{(AB)}$ varies as a function of a frequency tuning parameter, $\Delta \upsilon$. In Fig. 8, we plot the phases ${\varphi }_{12}^{\left(A\right)}(\Delta \upsilon )$, ${\varphi }_{13}^{\left(A\right)}(\Delta \upsilon )$, ${\varphi }_{12}^{\left(B\right)}(\Delta \upsilon )$ and ${\varphi }_{13}^{\left(B\right)}(\Delta \upsilon )$ reported in Figs. 3 and 4 over a narrow range of $\Delta \upsilon$ that encompasses a single π jump in ${\varphi }_{12}^{\left(B\right)}$. We recall that these phases of logical phi-bits are nonlinearly correlated as they depend on the same external driving conditions. This corresponds to regions of absolute frequencies in Figs. 3 and 4 centered on 172* kHz* for phi-bit A and 110* kHz* for phi-bit B. We now consider a change of frequency tuning parameter from ${\Delta \upsilon }_{2}$ to ${\Delta \upsilon }_{1}$ leads to very small changes in ${\varphi }_{12}^{\left(A\right)}$, ${\varphi }_{13}^{\left(A\right)}$, and ${\varphi }_{13}^{\left(B\right)}$. In contrast ${\varphi }_{12}^{\left(B\right)}$ undergoes a π jump. The state vector ${\Phi }^{(AB)}$ transforms approximately as

45 $$\frac{1}{2\sqrt{2}}\left(\begin{array}{c}{e}^{i{\varphi }_{12}^{(A)}}+{e}^{i{\varphi }_{12}^{(B)}}\\ {e}^{i{\varphi }_{12}^{(A)}}-{e}^{i{\varphi }_{12}^{(B)}}\\ {e}^{i{\varphi }_{13}^{(A)}}+{e}^{i{\varphi }_{13}^{(B)}}\\ {e}^{i{\varphi }_{13}^{(A)}}-{e}^{i{\varphi }_{13}^{(B)}}\end{array}\right)\to \frac{1}{2\sqrt{2}}\left(\begin{array}{c}{e}^{i{\varphi }_{12}^{(A)}}-{e}^{i{\varphi }_{12}^{(B)}}\\ {e}^{i{\varphi }_{12}^{(A)}}+{e}^{i{\varphi }_{12}^{(B)}}\\ {e}^{i{\varphi }_{13}^{(A)}}+{e}^{i{\varphi }_{13}^{(B)}}\\ {e}^{i{\varphi }_{13}^{(A)}}-{e}^{i{\varphi }_{13}^{(B)}}\end{array}\right)$$

Here, the first two complex amplitudes are permuted by the effect of adding π to ${\varphi }_{12}^{(B)}$ and keeping all other phases constants. This is the action of the controlled NOT gate that can be represented by the matrix $$\left(\begin{array}{cccc}0& 1& 0& 0\\ 1& 0& 0& 0\\ 0& 0& 1& 0\\ 0& 0& 0& 1\end{array}\right)$$. Note that this matrix has operated on complex amplitude coefficients. By choosing that the interval ${[\Delta \upsilon }_{2},{\Delta \upsilon }_{1}]$ encompasses an eventual hysteresis, this gate operation is reversible. This is but one example of how to realize quantum-like logic gates using logical phi-bits.

The generalization of the notion of representations of multiple (*N* > 2) correlated phi-bits as 2^* N*^ dimensional vector states and the exploitation of the background phases as well as nonlinear phase jumps as functions of the frequency tuning parameter may enable operations in exponentially complex Hilbert spaces.

## Conclusions

We have measured experimentally and analyzed theoretically the behavior of logical phi-bits supported by a nonlinear system composed of an array of three waveguide that is driven with two different frequencies. Logical phi-bits are realized as the nonlinear modes with frequencies that are linear combinations of the driving frequencies. The coherent state of phi-bits, analogous to that of a qubit, is characterized by the phase difference between waveguides measured at the end of the waveguides. A phi-bit state lives in a 2D Hilbert space and spans the Bloch sphere. We have shown that by tuning the frequency of one of two drivers, one can change the relative phase of the waveguides and therefore navigate the phi-bit Hilbert space. The changes in phase between the waveguides exhibits a background which variations scales as thousands of Hz. This background is shown theoretically to possibly originate from extrinsic effects associated with the nonlinearity of the electronics/transducer/ultrasonic-couplant assembly that enables the driving and characterization of the physical system. This background takes the form of a linear superposition of the phases associated with the vibrational modes at the primary driving frequencies. One can therefore easily subtract this background from the experimentally measured phi-bit phases to isolate the intrinsic behavior of phi-bits. The phi-bit phases also show dramatic jumps which occur very narrow ranges of driving frequency. The phase change associated with these jumps is on the order of 180° but may vary in magnitude. These jumps also exhibit hysteresis dependent on the direction of tuning, ascending or descending, of the driving frequency. These phase variations are shown theoretically to possibly arise from intrinsic nonlinearity associated with the epoxy used to couple the waveguides along their length.

We can therefore exploit these phase jumps to achieve predictably single-bit or two-bit quantum-like operations or even quantum-like algorithms^6,16^. The phi-bits supported by the nonlinear array of waveguides are correlated. In quantum systems, correlation arises from the nonlinearity of the connection between the probability for measuring a state (the square of the wave function) and the wave function. However, since the quantum wave function is a probability amplitude, quantum computing with multiple qubits suffers from the fragility of quantum superpositions of states through the collapse of the wave function upon a perturbation such as a measurement. Here, in contrast, the intrinsic nonlinearity of the physical systems correlates the complex amplitudes (i.e., magnitude and phases) of multiple phi-bits. This classical nonlinear correlation enables us to go beyond navigating the Hilbert space of individual phi-bit by exploring multiple phi-bit superpositions of states in exponentially complex Hilbert spaces. By manipulating the state of correlated phi-bits one may be able to seek single, large unitary transformations on multiple phi-bits that can operate predictably as composites of the more conventional operations on one or two bits. The present work is therefore a step forward toward the development of decoherence-free, measurement-able, predictable, operable, correlated phi-bits—analogous to qubits—as core components for practical QIS technologies that do not suffer from quantum fragility. Future work includes establishing new correspondences between the physical state of more than two correlated logical phi-bits and representations as complex vector in high-dimensional, exponentially scaling Hilbert space. These representations will enable experimental implementation of a nontrivial multi phi-bit unitary operation analogous to quantum circuits by a small number of simple actions of the state of the physical system such as frequency tuning; a very challenging task for current quantum computers.

## Data Availability

The datasets used and/or analysed during the current study are available from the corresponding author on reasonable request.
